# 14–3-3ζ inhibits heme oxygenase-1 (HO-1) degradation and promotes hepatocellular carcinoma proliferation: involvement of STAT3 signaling

**DOI:** 10.1186/s13046-018-1007-9

**Published:** 2019-01-03

**Authors:** Jia Song, Xiaochao Zhang, Zhibin Liao, Huifang Liang, Liang Chu, Wei Dong, Xuewu Zhang, Qianyun Ge, Qiumeng Liu, Pan Fan, Zhanguo Zhang, Bixiang Zhang

**Affiliations:** 1 Hepatic Surgery Center, Tongji Hospital, Tongji Medical College, Huazhong University of Science and Technology, Wuhan, 430030 China; 2 Hubei Province for the Clinical Medicine Research Center of Hepatic Surgery, Wuhan, 430030 China; 3 Key Laboratory of Organ Transplantation, Ministry of Education and Ministry of Public Health, Wuhan, 430030 China

**Keywords:** 14–3-3ζ, HO-1, Proliferation, STAT3, Hepatocellular carcinoma

## Abstract

**Background:**

Heme oxygenase 1 (HO-1) has been reported to be very important in the pathogenesis or progression of multiple types of cancer. Identification of novel hmox1 binding proteins may reveal undefined oncogenes, tumor suppressors, signaling pathways, and possible treatment targets.

**Methods:**

Immunoprecipitation and mass spectrometry analyses were used to identify novel regulators of HO-1. The association of the 14–3-3ζ protein with HO-1 and modulation of the stability of HO-1 were investigated by co-immunoprecipitation, immunofluorescence, western blotting, and quantitative RT-PCR. Degradation and in vivo ubiquitination assays were utilized to examine whether 14–3-3ζ stabilizes the HO-1 protein by inhibiting its ubiquitination. The effect of 14–3-3ζ on proliferation was investigated by function assays conducted in vitro using the CCK-8 and colony formation assays and in vivo in a xenograft mouse model. The biological functions of the 14–3-3ζ/HO-1 axis were demonstrated by western blotting and rescue experiments. Using gain-of-function and loss-of-function strategies, we further clarified the impact of 14–3-3ζ/HO-1 complex on the signal transducers and activators of transcription 3 (STAT3) signaling pathway in cancer cells.

**Results:**

We identified 14–3-3ζ as a novel HO-1 binding protein. The binding inhibited the ubiquitination and proteasome-mediated degradation of HO-1, thus facilitating its stabilization. Enforced expression of 14–3-3ζ significantly promoted cell proliferation in vitro, as well as tumorigenesis in vivo, while 14–3-3ζ knockdown had opposite effects. The data indicated that 14–3-3ζ can stabilize HO-1 expression and thus influence cancer cell proliferation. We further demonstrated the involvement of the STAT3 pathway in 14–3-3ζ/HO-1 regulation of hepatocellular carcinoma cell proliferation.

**Conclusions:**

Collectively, these data show that 14–3-3ζ regulates the stability of HO-1 to promote cancer cell proliferation and STAT3 signaling activation. The data establish the 14–3-3ζ-HO-1-STAT3 axis as an important regulatory mechanism of cancer cell growth and implicate HO-1 and 14–3-3ζ as potential therapeutic targets in hepatocellular carcinoma.

**Electronic supplementary material:**

The online version of this article (10.1186/s13046-018-1007-9) contains supplementary material, which is available to authorized users.

## Background

Heme oxygenase-1 (HO-1) is a stress-inducible, intracellular, rate-limiting enzyme that catalyzes the conversion of heme to biliverdin, carbon monoxide, and ferrous iron [1]. HO-1 and its enzymatic products are important in maintaining cellular homeostasis and regulate important biological processes including oxidative stress, inflammation, apoptosis, cell proliferation, fibrosis, and angiogenesis [2]. Due to the potential multiple effects of HO-1 in diverse conditions, HO-1 has been implicated in a wide spectrum of human diseases, including malignancies [3]. Several lines of evidence have highlighted the role of HO-1 in cancer progression and its expression correlates with tumor growth, aggressiveness, metastatic and angiogenetic potential, resistance to therapy, and poor prognosis [4]. Overexpression of HO-1 is closely related to cancer cell proliferation and downregulating HO-1 expression inhibits cell proliferation in many tumors [5].

The roles of the HO-1 protein in cellular signaling, including binding to proteins, phosphorylation, and modulation of protein function, among others, is being clarified [6]. The ubiquitination of HO-1 might be involved in its endoplasmic reticulum (ER)-associated proteasomal degradation. Thus, the ubiquitination plays a role in the stability of this protein [7]. Lin et al. reported that translocation in renal carcinoma, chromosome 8 (TRC8) forms a complex with HO-1, and identified TRC8 as an E3 ligase involved in the ubiquitination of HO-1 [8]. Salinas et al. demonstrated that protein kinase AKT/PKB can phosphorylate recombinant human HO-1 in vitro and in HEK293T cells at the Ser188 residue [9]. A large body of evidence has indicated that post-translational alterations and modifications of HO-1 may correlate with the development of cancer, including cancer cell proliferation and tumor metastasis [1]. Thus, a more complete understanding of HO-1 modifications and the properties that they impart is needed, and a focus on the protein interactions of HO-1 could lead to clinical interventions that prevent tumor progression and cell proliferation.

The 14–3-3 family of seven proteins (β, γ, ε, σ, ζ, τ, and η) is highly conserved in all eukaryotic cells. These proteins interact with a diverse array of cellular proteins, including transcription factors, biosynthetic enzymes, cytoskeletal proteins, signaling molecules, and apoptosis factors [10]. By interacting with its partners, 14–3-3 proteins affect various physical and functional aspects of these proteins, such as binding to other proteins, subcellular distribution, stability, and catalytic activity, and can serve as an adaptor/scaffold protein [11–13]. The involvement of 14–3-3 proteins in a myriad of cellular processes has been demonstrated, including cell cycle control, growth, differentiation, apoptosis, and mitogenic or stress signaling [13, 14]. The ability of 14–3-3 proteins to bind and regulate various oncogenic gene products as well as various tumor suppressor gene products points to their potentially key role in cancer [15].

The ζ isoform of 14–3-3 is a potential prognostic and therapeutic target protein [16]. It has been implicated in the initiation and progression of cancer and is overexpressed in multiple cancer types, including breast cancer [17], lung cancer [18], colorectal carcinoma [19], and hepatocellular carcinoma (HCC) [20]. The elevated expression of 14–3-3ζ has been correlated with cancer progression and poor prognosis [16, 21]. The overexpression of 14–3-3ζ in multiple types of cancers and its significant association with poor prognosis highlight the need to target 14–3-3ζ. The development of novel 14–3-3ζ–targeting agents/effectors is an underdeveloped area of study, but is important to pursue since it could lead to therapeutic strategies for HCC.

In this study, to better understand the molecular events involved in the hmox1-mediated signaling pathway, we used a combined immunoprecipitation/mass spectrometry (IP/MS) approach to identify novel hmox1-mediated protein-protein interactions. The data show that 14–3-3ζ, interacts with HO-1 and regulates HO-1 stability by inhibiting its ubiquitination. Thus, 14–3-3ζ influence cancer cell proliferation. The findings also demonstrate crucial roles for the 14–3-3ζ/HO-1 complex in the regulation of STAT3 signaling and cancer cell survival.

## Methods

### Cell lines and culture conditions

The HCC cell lines HLF, SK-Hep1, and Bel7402 were obtained from the Hepatic Surgery Center, Tongji Hospital, Huazhong University of Science and Technology, Wuhan, China. HEK293 cells were purchased from the China Center for Type Culture Collection (Wuhan, China). The cell lines were maintained in Dulbecco’s Modified Eagle’s Medium (DMEM) supplemented with 10% fetal bovine serum, 100 U/mL penicillin, and 0.1 mg/mL streptomycin in a 5% CO_2_ incubator at 37 °C.

### Plasmids

To establish hmox1 or 14–3-3ζ overexpressing cell lines, the human cDNA was cloned into the pBABE-puro retroviral vector (plasmid 1765, Addgene, Cambridge, MA, USA). To establish stable 14–3-3ζ, hmox1, and STAT3 knockdown or control cells, the target short hairpin RNA (shRNA) sequences and one non-targeting sequence (negative control, NC) were selected and cloned into the pLKO.1 vector (pLKO.1 puro, plasmid #8453, Addgene). Viral packaging and transduction were performed as previously described [22]. The target shRNA sequences are listed in Additional file 1: Table S1. Mammalian expression plasmids (pcDNA3.1) for Flag- or HA-tagged HO-1 and its mutants, HA-tagged 14–3-3ζ were created according to a standard PCR-based targeted mutagenesis protocol and were verified by sequencing (Sangon Biotech Co., Ltd., Shanghai, China). The sequences of the primer pairs used to generate each mutant are listed in Additional file 1: Table S2. STAT3 luciferase reporter plasmids were purchased from Qiagen. Mammalian expression plasmids for JAK1, and its mutants, JAK2 and its mutants, STAT3 and its mutants were constructed by standard molecular biology techniques and purchased from GeneChem (Shanghai, China).

### Mass spectrometry (MS)

Whole cell lysates from 293FT cells transient transfected with FLAG-HO-1 or FLAG-vector were lysed in lysis buffer (50 mM Tris-HCl (pH 7.4), 100 mM NaCl, 0.5% NP-40, 1 mM EDTA and protease inhibitor cocktails) and applied to an IP lysis buffer equilibrated FLAG affinity gel (Sigma-Aldrich, St. Louis, MO, USA) in 4 °C for 4 h. After binding, the affinity gels were washed with the lysis buffer for five times and the pellets were resuspended in SDS sample buffer and boiled for 8 min. The final eluted proteins were separated by SDS-PAGE followed by silver staining of the gel. There was a great difference in the bands between the FLAG-HO-1 group and the control FLAG-vector group among the molecular weight 15kd-40kd region of the gel. The relevant bands (from 15kd~40kd) were excised from the gel and cut into pieces. For in-gel tryptic digestion, gel pieces were destained in 50 mM NH4HCO3 in 50% acetonitrile (*v*/v) until clear. Gel pieces were dehydrated with 100 μl of 100% acetonitrile for 5 min, the liquid removed, and the gel pieces rehydrated in 10 mM dithiothreitol and incubated at 56 °C for 60 min. Gel pieces were again dehydrated in 100% acetonitrile, liquid was removed and gel pieces were rehydrated with 55 mM iodoacetamide. Samples were incubated at room temperature, in the dark for 45 min. Gel pieces were washed with 50 mM NH4HCO3 and dehydrated with 100% acetonitrile. Gel pieces were rehydrated with 10 ng/μl trypsin resuspended in 50 mM NH4HCO3 on ice for 1 h. Excess liquid was removed and gel pieces were digested with trypsin at 37 °C overnight. Peptides were extracted with 50% acetonitrile/5% formic acid, followed by 100% acetonitrile. Peptides were dried to completion and resuspended in 2% acetonitrile/0.1% formic acid. Then, the resulting tryptic peptides were subjected to liquid chromatography-tandem MS (LC-MS/MS) sequencing. The masses of peptides were identified by time-of-flight analysis using a model 4700 protein analyzer (Applied Biosystems, Franklin Lakes, NJ, USA). Data obtained by MS and MS/MS were queried against the Swiss-Prot database (*Homo sapiens*).

### Immunofluorescent staining

For immunofluorescent microscopy after the indicated treatments, cells were seeded onto glass slides for 24 h, fixed in 4% paraformaldehyde, and permeabilized with 0.5% Triton X-100 for 15 min. The slides were then incubated with a primary antibody in blocking solution overnight at 4 °C in a humidified chamber. The slides were then washed three times with phosphate buffered saline (PBS) and incubated with one or both of two secondary antibodies (from different species) for 1 h at room temperature in a humidified chamber. Finally, the coverslips were incubated with 40, 60-diamidino-2-phenylindole (Sigma-Aldrich) for 15 min, and images were obtained by phase-contrast and confocal microscopy.

### Immunoblotting, co-immunoprecipitation (co-IP) and protein ubiquitination assays

Immunoblot, co-IP, and in vivo ubiquitination assay were carried out as described previously [22].

### Cell proliferation assay and colony formation assay

Cell proliferation was measured using the Cell Counting Kit-8 (CCK-8, Beyotime Institute of Biotechnology, Shanghai, China). Cells were plated in 96-well plates at the indicated density and cultured in complete medium. At the indicated times, 10 μL of CCK-8 was added to 90 μL of culture medium per well for 2 h at 37 °C, and the plate was read using an Elx 800 enzyme-linked immunosorbent assay plate reader (Bio-Tek, Winooski, VT, USA) at 450 nm. Each cell line was assayed in triplicate wells, and the experiment was repeated three times. For the colony formation assay, 1000 to 3000 cells were plated into each well of a 6-well plate and incubated at 37 °C for 2 weeks. The colonies were fixed and stained with a dye solution containing 0.1% crystal violet and 20% methanol, and the number of colonies was counted. All assays were replicated three times.

### Transfection and luciferase reporter assays

Small interfering RNA (siRNA) and si-control were purchased from Ribobio (Guangzhou, China). Lipofectamine 3000 (Invitrogen, Carlsbad, CA, USA) was used for the transient transfection of small RNA oligos and plasmids according to the manufacturer’s instructions. siRNA target sequences are listed in Additional file 1: Table S3. Luciferase assays were performed using a dual-specific luciferase assay kit (Promega) according to manufacturer’s protocols after transfection. All reporter assays were repeated at least three times.

### Quantitative real-time PCR assay

Total RNA was extracted using TRIzol Reagent (Life Technologies, Carlsbad, CA, USA) and reverse transcription was performed using the PrimeScript® RT reagent Kit (Takara Bio, Dalian, China) according to the manufacturer’s protocol. Quantitative real-time PCR was carried out using the CFX96 Touch™ Real-Time PCR Detection System (Bio-Rad, Hercules, CA, USA) using the SYBR Green Supermix kit (Takara Bio) according to the manufacturers’ instructions. All of the gene expression levels were normalized to that of the housekeeping gene glyceraldehyde-3-phosphate dehydrogenase (GAPDH). Each reaction was repeated independently at least three times in triplicate. The primers used are summarized in Additional file 1: Table S4.

### Xenograft study

Male Balb/c athymic nude mice (3–4-week-old) were housed under specific pathogen-free conditions and cared for according to the institutional guidelines on animal care. The mice were randomly divided into the indicated groups (5–8 mice/group) before inoculation. For subcutaneous injection, 2 × 10^6^ tumor cells were suspended in 100 μL of serum-free DMEM, and then injected subcutaneously into the flank of the nude mice. Tumor development in the mice was observed every 3 days. The mice were sacrificed at a defined endpoint. Tumors were removed, photographed, measured, and weighed. The average volume and weight of the tumors were calculated.

### Cell viability assay, AnnexinV/Propidium iodide staining, and TUNEL assay

Cell viability was analyzed by Trypan Blue Staining Cell Viability Assay Kit (Beyotime Institute of Biotechnology, Shanghai, China) and counted by Nexcelom Cellometer Auto X4 Cell Counter (Nexcelom Bioscience, Massachusetts, USA). All viability experiments were repeated as three independent experiments, and mean percentage of cell survival was calculated along with SD. Student’s t test was used for calculating statistical significance. Cell apoptosis was done by Annexin V-fluorescein isothiocyanate (FITC)/propidiumiodide (PI) double staining using flow cytometry analysis. Cells were incubated with Annexin V-FITC and PI for 15 min at room temperature in the dark. The samples were processed to the FASCcan Flow Cytometer (Becton-Dickinson, Franklin Lakes, NJ, USA). Apoptosis in tumor was examined by TUNEL assay using TUNEL Apoptosis Assay Kit (Beyotime Institute of Biotechnology, Shanghai, China). Tissue sections were dewaxed and rehydrated according to standard protocols. TUNEL staining was performed according to the protocols from the manufacturers. Apoptotic cells were photographed with microscope.

### Reagents and antibodies

Cycloheximide and MG132 were purchased from Calbiochem (San Diego, CA, USA). Antibodies were obtained from the following sources: monoclonal anti-HO-1 (ab52947, Abcam, Cambridge, UK; used for Immunoblot, co-IP), polyclonal anti-HO-1 (10701–1-AP, Proteintech, Chicago, IL, USA; used for Immunofluorescent staining), anti-β-actin (A5316, Sigma-Aldrich), monoclonal anti-Flag (F1804, Sigma-Aldrich), monoclonal anti-HA (H9658, Sigma-Aldrich), polyclonal anti-Flag (F7425, Sigma-Aldrich), anti-14-3-3β (sc-25,276, Santa Cruz Biotechnology, Dallas, TX, USA), anti-14-3-3θ (sc-59,414 Santa Cruz Biotechnology,), anti-14-3-3η (sc-423,749, Santa Cruz Biotechnology), anti-14-3-3γ (12381-1AP, Proteintech), anti-14-3-3ε (11648–2-AP, Proteintech), anti-14-3-3ζ (15222–1-AP, Proteintech), anti-ERp72(66365–1-Ig, Proteintech), anti-STAT3 (#12640, Cell Signaling Technology, Beverly, MA, USA), anti-JAK2 (#3230, Cell Signaling Technology), anti-pY705-STAT3 (#9145, Cell Signaling Technology), anti-pS727-STAT3 (#9134, Cell Signaling Technology), and and anti-ubiquitin (#3936, Cell Signaling Technology). Fluorochrome-conjugated secondary antibodies were from Thermo Fisher Scientific (Waltham, MA, USA). Agarose-immobilized anti-FLAG (M2, Sigma-Aldrich) was used for IP. The antibodies used as negative controls for IP included normal mouse IgG (sc-2025, Santa Cruz Biotechnology) and normal rabbit IgG (sc-2027, Santa Cruz Biotechnology).

### Statistical analyses

Data were statistically analyzed using Graphpad Prism 6 software (GraphPad Software, San Diego, CA, USA), and presented as mean ± s.d., Unpaired two-tailed t-test or ANOVA test was utilized to analyze the difference between the two groups. Three independent experiments were performed to guarantee reproducibility of findings. A *P*-value < 0.05 was considered statistically significant.

## Results

### Identification of 14–3-3ζ as a binding partner for HO-1

**Fig. 1:**
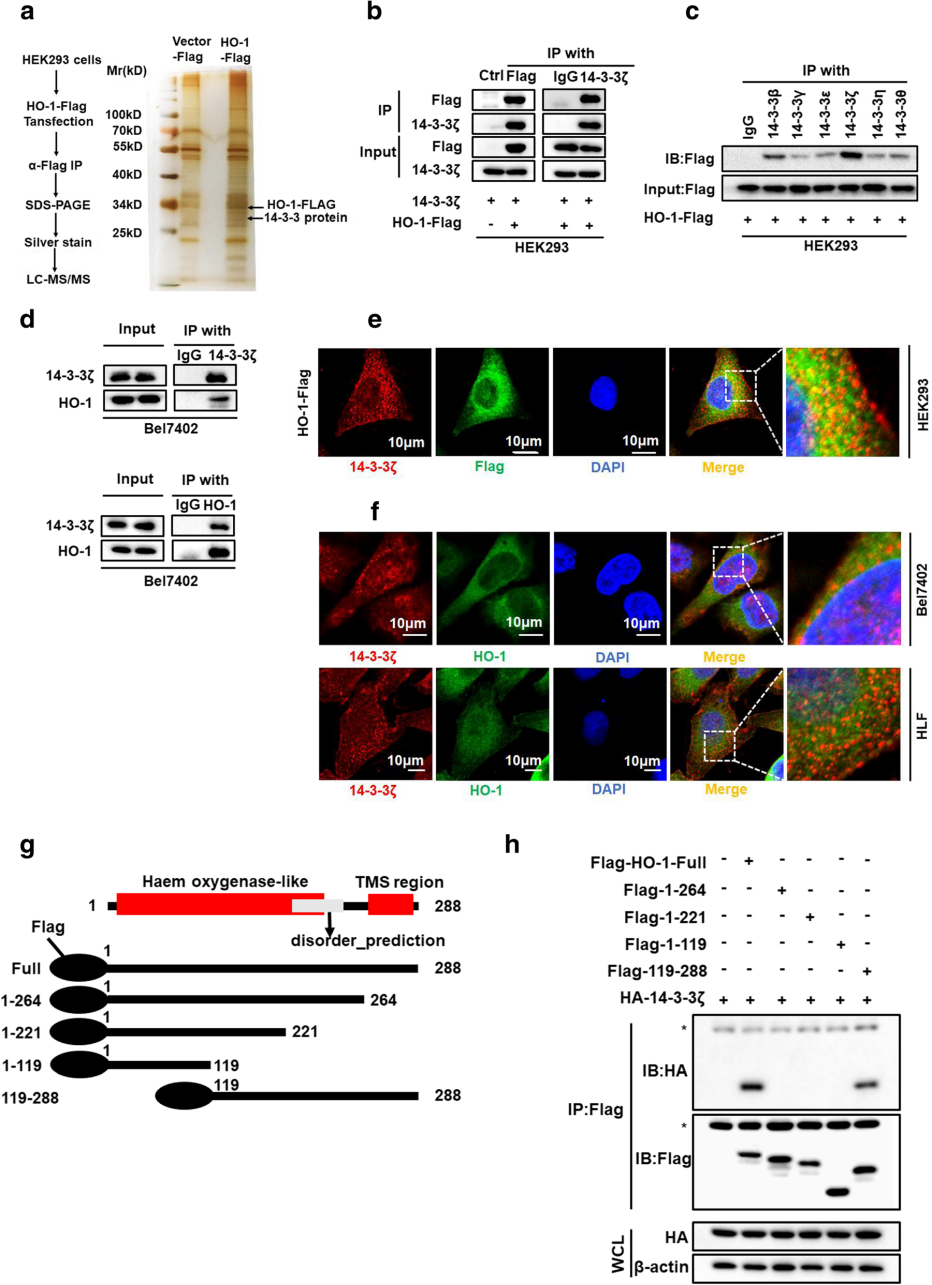
Identification of 14–3-3ζ as a binding partner for HO-1 (**a**) MS analysis of HO-1-associated proteins. **b** Whole cell lysates from HEK293 cell transfected with Flag-tagged HO-1 were subjected to co-IP with anti-FLAG or anti-14-3-3ζ antibodies and followed by immunoblotting with antibody against FLAG, 14–3-3ζ. **c** HEK293 cells were transfected with a plasmid encoding Flag-tagged HO-1. The lysates were immunoprecipitated with anti-14-3-3 antibodies against the indicated 14–3-3 isoforms, and were then analyzed by western blotting. **d** Bel7402 cell lysate was subjected to co-IP with anti-14-3-3ζ or anti-HO-1, followed by immunoblotting with antibody against 14–3-3ζ, HO-1. **e** HEK293 cells were transfected with Flag-tagged HO-1. After 24 h of transfection, immunofluorescence staining was performed to observe the co-localization of exogenously expressed HO-1 and 14–3-3ζ. Scale bar denotes 50 μm. **f** Confocal experiments were performed to determine the co-localization between endogenous HO-1 and 14–3-3ζ. Scale bar denotes 10 μm. **g** Schematic diagram of FLAG-tagged full-length or deletion constructs of HO-1 used in this study. **h** Whole cell lysate from HEK293 cell transfected with the indicated plasmids were subjected to co-IP with anti-FLAG and followed by immunoblotting with indicated antibodies. The asterisk denotes a nonspecific band. WCL denotes whole-cell lysate

To better understand the molecular events involved in hmox1-mediated signaling, we used a combined IP/MS approach to identify novel HO-1-mediated protein-protein interactions. The total cell extracts prepared from HEK293 cells overexpressing FLAG-tag or FLAG-tagged HO-1 were immunoprecipitated and analyzed by gradient elution LC-MS/MS (Fig. 1a). Novel interactors were identified, including the 14–3-3 protein major isoform (Additional file 2: Figure S1a). Analysis of the MS data indicated that the 14–3-3 proteins were probable HO-1 interacting partners, considering that the 14–3-3 protein epsilon isoform was identified with two matching peptides, the 14–3-3 protein zeta isoform with four matching peptides, the 14–3-3 protein gamma isoform with one matching peptide, and the 14–3-3 protein beta/alpha isoform with one matching peptide (Additional file 2: Figure S1a and b). The finding of 14–3-3 protein in FLAG-HO-1 immunoprecipitates was of particular interest as it has been reported to be the master integrator of diverse signaling cues that influence cell fate decisions and tumorigenesis through protein binding [10]. To confirm the MS data, the immunoprecipitates were probed for 14–3-3ζ and FLAG-HO-1. The interactions of exogenous HO-1 with 14–3-3ζ was confirmed by co-IP in HEK293 cells (Fig. 1b). To determine whether other 14–3-3 isoforms could also interact with HO-1, 293FT cells transient transfected with FLAG-HO-1 were immunoprecipitated using 14–3-3 isoform specific antibodies and the co-precipitated FLAG-HO-1 was detected by Western blot. The results showed that other 14–3-3 isoforms could also interact with HO-1 (Fig. 1c).

We next tested for the presence of the 14–3-3ζ/HO-1 interaction under endogenous conditions. IP of 14–3-3ζ or HO-1 revealed the interaction of endogenously expressed 14–3-3ζ and HO-1 in HCC cells (Fig. 1d). Furthermore, immunofluorescence confocal microscopy revealed a high degree of spatial concordance between 14 and 3-3ζ and HO-1, suggesting that 14–3-3ζ and HO-1 co-localized in the Bel7402 and HLF cell lines (Fig. 1e and f). We also observed the subcellular localization of HO-1 and 14–3-3ζ in tumor cells. We monitored the localization of HO-1 and 14–3-3ζ in wild-type HCC cells by immunofluorescence, compared with ER marker protein. Confocal images were focused on the ER planes, to enhance co-localization. As shown in Additional file 3: Figure S2a and b, HO-1 and 14–3-3ζ localized primarily to an intracellular compartment, co-localizing with ERp72 protein (ER marker), suggesting that HO-1 and 14–3-3ζ were partially localized in ER.

The 14–3-3 family of proteins contains three high-affinity binding motifs: RSXpS/TXP (mode 1), RXXXpS/TXP (mode 2), and pS/T-X (1–2)-COOH (mode 3) [23]. Unfortunately, we did not find any canonical binding motifs in the HO-1 protein by analysis of the amino acid sequence of human HO-1. Recent studies have shown that 14–3-3 proteins can also bind to atypical motifs in some proteins [24]. Based on this information, we selected four possible predicted motif sites, which were close to these three well-characterized motifs according to available data (https://scansite4.mit.edu/4.0/). As a result of this analysis, we prepared four point mutations of HO-1, where either Ser or Thr residues, as potential phosphorylation sites, were replaced with an Ala residue (S8A, T124A, S188A, and S247A). These point mutants were tested for 14–3-3 binding using an assay similar to that described above (Additional file 3: Figure S2c). As shown in Additional file 3: Figure S2d, these mutations in HO-1 did not affect their interaction with 14–3-3 proteins in vitro. In addition to the phosphorylation sites, most 14–3-3 protein-binding motifs are within disordered regions [24, 25]. To identify the region of HO-1 responsible for the 14–3-3ζ interaction, we generated a series of Flag-tagged HO-1 deletion mutants and transfected them into HEK293 cells along with HA-tagged 14–3-3ζ (Fig. 1g). The region encompassing amino acids 265–288 in HO-1 was essential for the interaction with 14–3-3ζ (Fig. 1h). Collectively, these data demonstrate that 14–3-3ζ could associate with HO-1, and that the interaction was mediated through a disordered region of HO-1 rather than through phosphorylation of a motif.

### 14–3-3ζ stabilizes HO-1 protein expression by protecting HO-1 from ubiquitin-mediated degradation

**Fig. 2:**
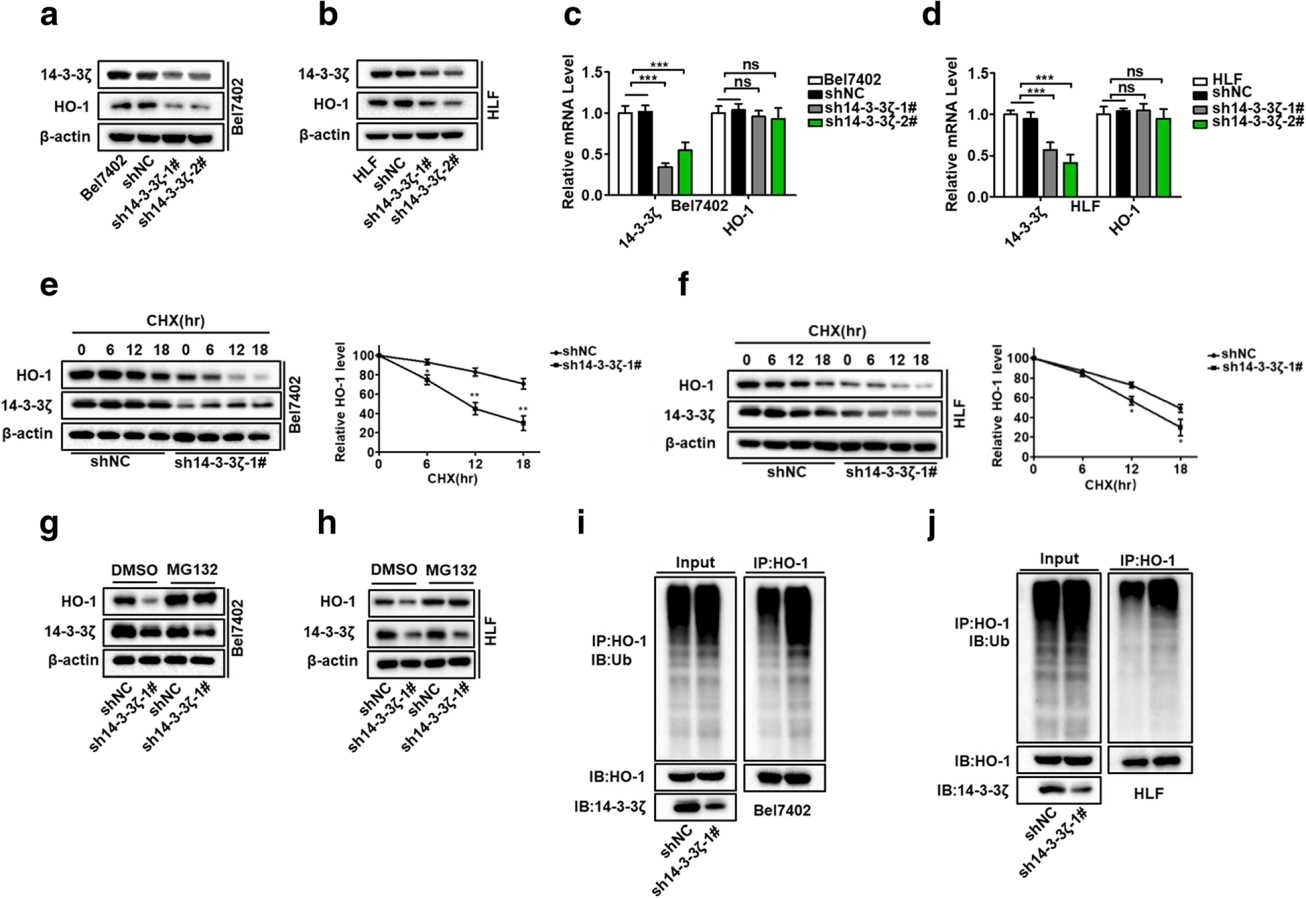
14–3-3ζ stabilizes HO-1 protein expression by protecting HO-1 from ubiquitin-mediated degradation (**a**, **b**) Western blot analysis of 14–3-3ζ and HO-1 protein levels in Bel7402 and HLF cell lines transfected with the sh14–3-3ζ plasmids. **c**, **d** qRT-PCR analysis of 14–3-3ζ and HO-1 mRNA levels after 14–3-3ζ knockdown. **e**, **f** 14–3-3ζ knockdown cells were treated with cycloheximide (CHX) for the indicated times and the expression of endogenous HO-1 protein was analyzed by western blotting (left panel). Three independent experiments were performed and results were quantified by ImageJ software and plotted (right panel). **g**, **h** 14–3-3ζ knockdown cells were treated with 20 μM MG132 or dimethylsulfoxide for 10 h. The cells were harvested and analyzed by western blotting with the indicated antibodies. **i**, **j** 14–3-3ζ knockdown cells were treated with 20 μM MG132 for 10 h, and the cell lysates were immunoprecipitated with an anti-HO-1 antibody followed by immunoblotting with an anti-ubiquitin antibody

The interaction between 14 and 3-3ζ and HO-1 prompted us to further investigate the relationship between these two proteins. We had observed that the knockdown of HO-1, as well HO-1 overexpression, in HCC cells had no effect on 14–4-3ζ expression at both the protein and mRNA levels (Additional file 4: Figure S3a, b, c, and d). We also investigated whether HO-1 affect the posttranscriptional stabilization of 14–3-3ζ. We examined the 14–4-3ζ protein levels in HLF HO-1 knockdown cells after treatment with cycloheximide to block newly protein synthesis. As expected, the degradation of 14–4-3ζ was not affected in the shHO-1 HCC cells compared with the control shNC cells, suggesting that HO-1 might have no effect on the regulating the posttranscriptional stability of 14–3-3ζ(Additional file 4: Figure S3e and f). Surprisingly, when co-expressed with HA-14-3-3ζ, Flag-HO-1 displayed a higher protein level in the co-transfected cells than when it was transfected alone but did not significantly affect the mRNA levels of Flag-HO-1 (Additional file 4: Figure S3g and h). To further confirm the stabilizing effect of 14–3-3ζ on HO-1, we knocked down 14–3-3ζ expression in HCC cells using a lentiviral-mediated shRNA. As expected, knockdown of 14–3-3ζ significantly reduced the protein expression of HO-1 (Fig. 2a and b) but had no effect on mRNA levels (Fig. 2c and d). The posttranscriptional stabilization of HO-1 by 14–3-3ζ suggested that the 14–3-3ζ protein can protect HO-1 from degradation. As predicted, knockdown of 14–3-3ζ expression significantly reduced the half-life of HO-1 after inhibiting new protein translation with cycloheximide (Fig. 2e and f). Moreover, the downregulation of HO-1 in 14–3-3ζ-knockdown cells was restored in the presence of the proteasome inhibitor MG132 (Fig. 2g and h), indicating that 14–3-3ζ-knockdown facilitated the degradation of HO-1.

The HO-1 protein undergoes ubiquitination and proteasome-mediated degradation through an ER-associated degradation pathway [7, 8]. Thus, we next investigated whether 14–3-3ζ could prevent the degradation of HO-1 by affecting its ubiquitination. We immunoprecipitated HO-1 from cells pretreated with MG132 and probed the immunoprecipitates with anti-ubiquitin antibodies. As shown in Fig. 2i and j, ubiquitination of HO-1 was increased in Bel7402 and HLF cells with a 14–3-3ζ knockdown, compared to control cells. To examine whether the effect of 14–3-3ζ on HO-1 stability is isoform specific, we first determined the expression levels of 14–3-3 isoforms in HCC cell lines using real-time PCR analyses. The result showed that 14–3-3ζ is one of the major 14–3-3 isoform expressed in these cell lines (Additional file 5: Figure S4a and b). Specific siRNAs were used to silence each 14–3-3 isoform expression in the HCC cell line HLF. As shown in Additional file 5: Figure S4c-f, siRNA-mediated knockdown of other 14–3-3 isoforms almost had no effect on HO-1 expression at both the protein and mRNA levels. This suggested that other 14–3-3 isoforms may be dispensable in the process of regulating HO-1 ubiquitination and degradation in our system. Taken together, these results indicate that 14–3-3ζ could stabilize HO-1 expression by protecting HO-1 from ubiquitination and subsequent proteasomal degradation.

### 14–3-3ζ promotes cell proliferation in vitro and tumor growth in vivo

**Fig. 3:**
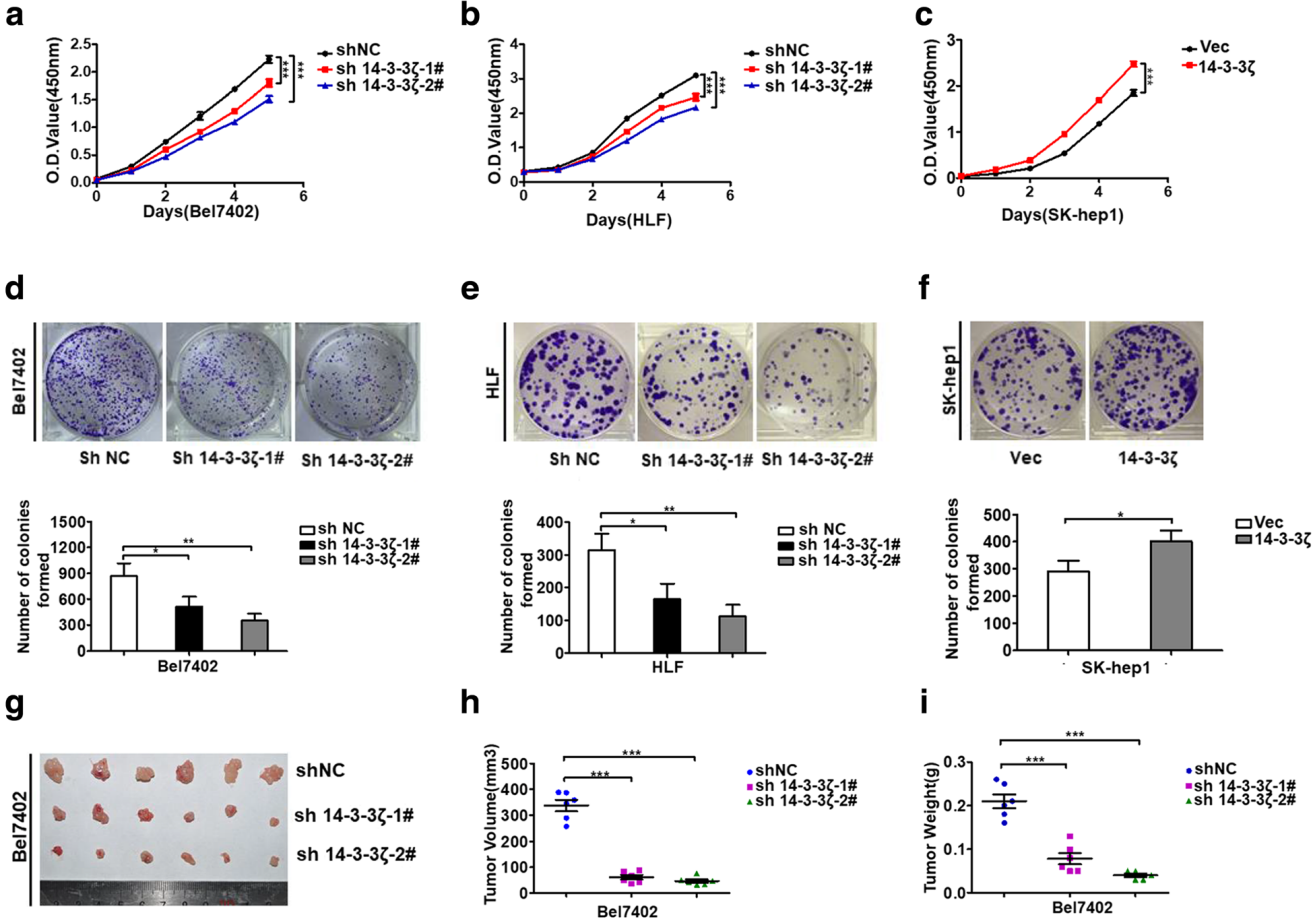
14–3-3ζ promotes cell proliferation in vitro and tumor growth in vivo (**a**-**c**) Cell proliferation was measured by the CCK-8 assay. ***P < 0.001 compared with corresponding NC or Vector. **d**-**f** Cell proliferation was examined by a colony formation assay, and relative clone number was used for statistic analysis. Data represent the mean ± SD of three independent experiments. *P*-values were calculated using the Student’s t-test. **g**-**i** 14–3-3ζ knockdown suppresses HCC tumor growth in vivo. **g**: Photos of the xenograft tumors; **h** Tumor volume; **i** Tumor weights. ***P < 0.001

Having established a role for 14–3-3ζ in stabilizing HO-1, we next sought to investigate the physiological function of this finding. The 14–3-3ζ protein has an oncogenic property in various types of cancer. To gain insight into the functional role of 14–3-3ζ in HCC cells, we examined the effect of 14–3-3ζ on cell growth and tumorigenicity. CCK-8 assays showed that knockdown of 14–3-3ζ expression significantly inhibited cell proliferation in Bel7402 and HLF cells compared to control cells (Fig. 3a and b). Similarly, results of colony-formation assays revealed that a decrease in 14–3-3ζ expression also greatly attenuated the colony-forming ability of Bel7402 and HLF cells (Fig. 3d and e). In contrast, overexpression of 14–3-3ζ promoted cell growth in SK-hep1 cells (Fig. 3c and f). To further assess the role of 14–3-3ζ in vivo, we used a xenograft mouse model. Bel7402 cells stably transfected with sh-14-3-3ζ or an empty vector were subcutaneously injected into nude mice. Up to 21 days after injection, tumors grown from 14 to 3-3ζ stable knockdown cells were smaller than tumors grown from control cells, and the average tumor weight in the sh14–3-3ζ group was markedly lower than that in the control group (Fig. 3g-i).

### HO-1 is implicated in 14–3-3ζ-mediated tumor proliferation

**Fig. 4:**
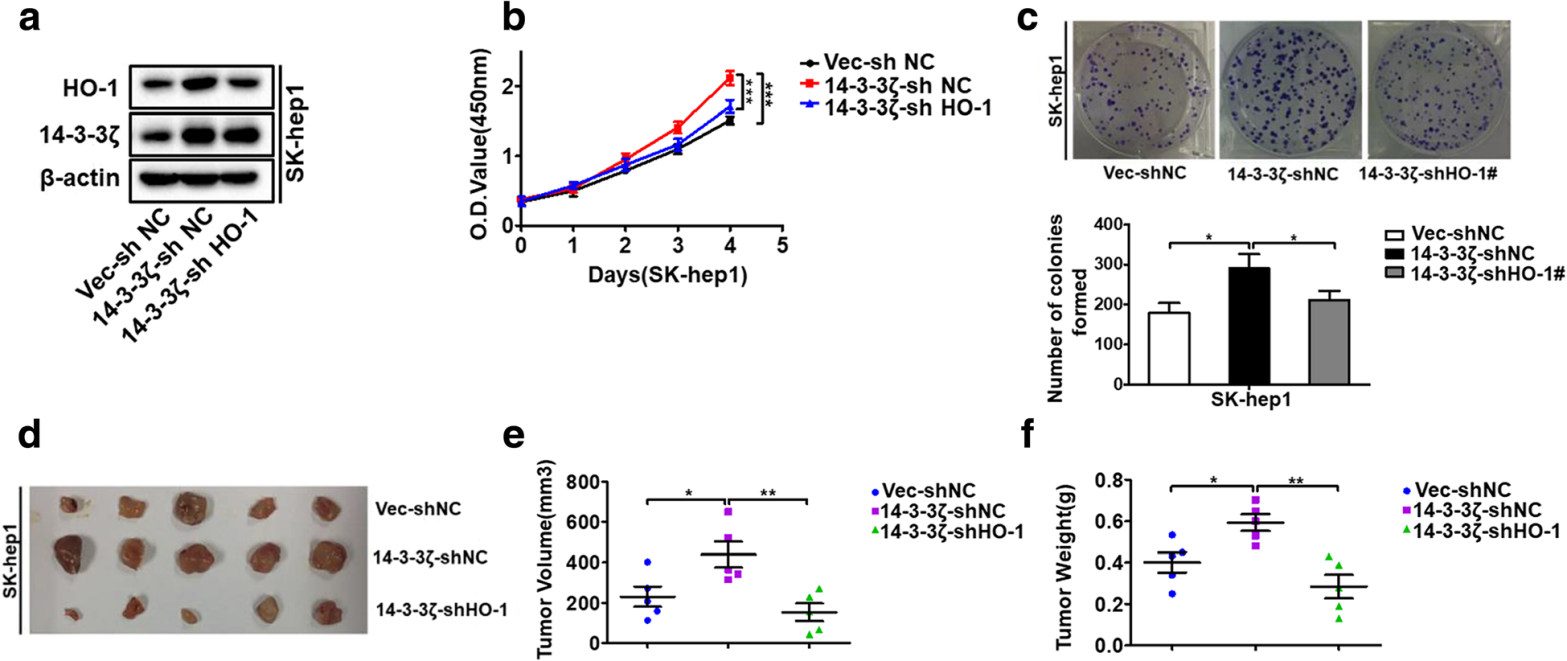
HO-1 is implicated in 14–3-3ζ-mediated tumor proliferation (**a**) 14–3-3ζ-overexpressing cells were stably transfected with shNC, or shHO-1 plasmids, to generate control (Vec-shNC) stable cells, 14–3-3ζ overexpressing (14–3-3ζ-shNC) stable cells, and 14–3-3ζ overexpressing but HO-1 knockdown (14–3-3ζ-shHO-1) stable cells. The expressions of HO-1 and 14–3-3ζ were examined by western blot analysis. **b** and **c** CCK8 assay and colony formation assay were performed for the indicated cells. **d**-**f** The indicated cells were subjected to an in vivo xenograft assay and tumor growth was assessed. **d** Photos of the xenograft tumors; **e** Tumor volume; **f** Tumor weights

HO-1 expression correlates with cancer growth and resistance to therapy as shown in different types of tumors, such as human renal cell carcinoma [26], prostate and pancreatic cancers [27, 28], melanoma [29], and hepatoma [30]. As we found that 14–3-3ζ could regulate HO-1 expression, we wondered if the pro-tumorigenic effects of 14–3-3ζ were mediated by HO-1. To test this, the effect of 14–3-3ζ on proliferation was analyzed in HCC cells with or without HO-1 knockdown (Fig. 4a). According to the growth curve and CCK-8 assay results, overexpression 14–3-3ζ significantly increased HCC cell growth, whereas HO-1 knockdown attenuated the effect of 14–3-3ζ on cell growth (Fig. 4b). Colony formation assay further confirmed this process (Fig. 4c). To determine whether these results were reproducible in vivo, we constructed a xenograft HCC model in nude mice. Consistent with the results from in vitro assays, 14–3-3ζ overexpression-induced tumor growth was effectively abrogated by HO-1 knockdown compared with the control (Fig. 4d-f). The collective results indicated that 14–3-3ζ promotes HCC cell growth by maintaining elevated levels of HO-1 in vitro and in vivo. Since the obligate compensatory suppression of apoptosis together with deregulated cell proliferation propelled the tumor cell and its progeny into uncontrolled expansion [31], we also detected the effect of 14–3-3ζ/HO-1 on HCC cell apoptosis by trypan blue exclusion assay and FACS-based Annexin V/PI staining. The results revealed that knockdown of 14–3-3ζ or over-expression of 14–3-3ζ did not result in measurable changes in the apoptotic states of HCC cells in vitro under normal culture conditions (Additional file 6: Figure S5a-d). TUNEL staining of HCC xenograft tumors derived from shNC and sh14–3-3ζ cells showed similar trends that there is no significant difference in the number of TUNEL+ cells between these two groups (Additional file 6: Figure S5e and f). These indicated that 14–3-3ζ might have no apoptosis-protecting effect in HCC cells, at least under normal culture conditions. We also investigated the effects of HO-1 on apoptosis of HCC cells. Trypan blue exclusion assay and flow cytometric analysis using Annexin V kit showed that apoptosis was not obviously affected in shHO-1-transfected HLF cells compared with control cells under normal culture conditions (Additional file 6: Figure S5 g and h). These results indicate that the promoted effect of cell proliferation by 14–3-3ζ/HO-1 may be not likely mediated by apoptosis in our system.

### 14–3-3ζ/HO-1 complex can regulate STAT3 signaling

**Fig. 5:**
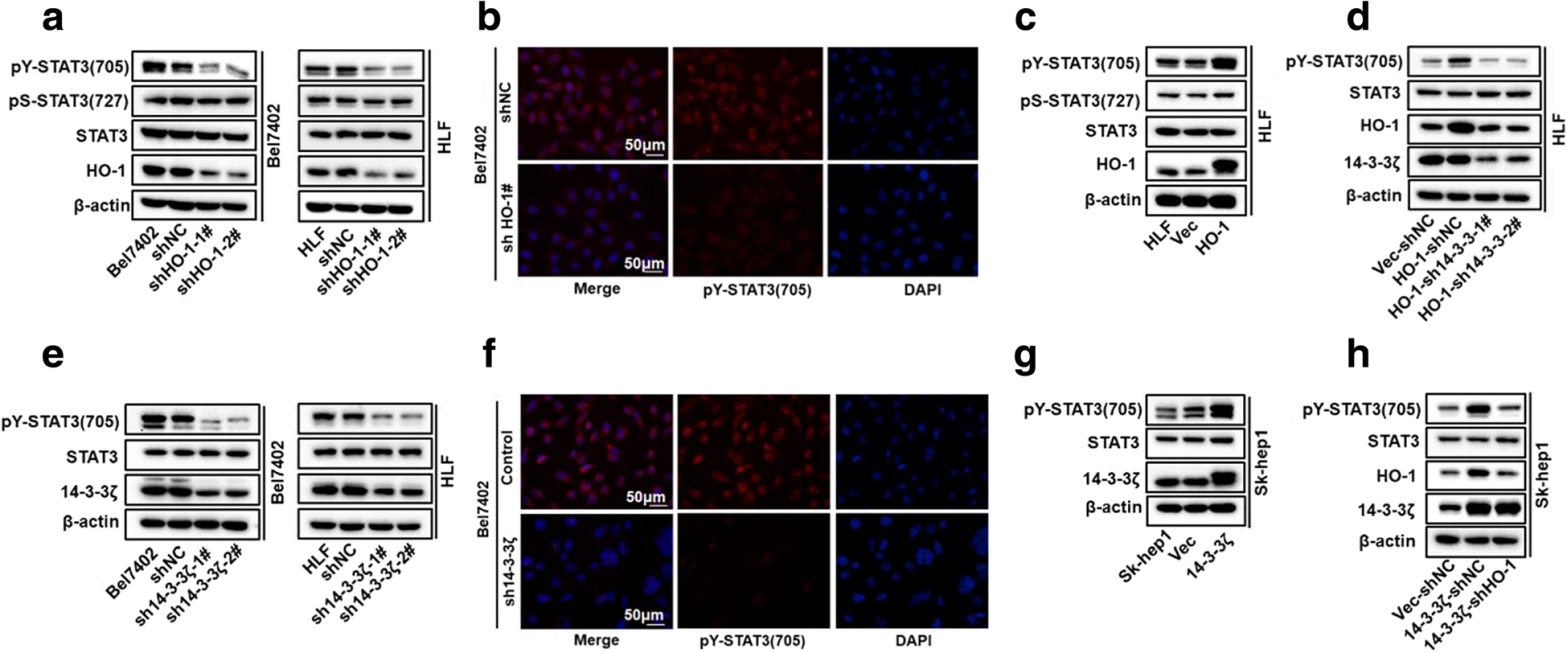
14–3-3ζ/HO-1 complex could regulate STAT3 signaling (**a** and **c**) p-STAT3 (Ser-727 and Tyr-705) and total STAT3 levels in the HO-1 knockdown cells (**a**) and HO-1-overexpressing cells (**c**) were determined by western blotting. **b** Immunofluorescent staining of pY-STAT3 in HO-1 knockdown cells. 4′,6-Diamidino-2-phenylindole dihydrochloride staining of the nucleus was done (blue); scale bar denotes 50 μm. **d** HO-1-overexpressing cells were stably transfected with shNC or sh14–3-3ζ plasmids. pY-STAT3 levels in the indicated cells were analyzed by western blotting. **e** and **g** pY-STAT3 levels in 14–3-3ζ knockdown cells (**d**) and 14–3-3ζ-overexpressing cells (**e**) were analyzed by western blotting. **f** Immunofluorescent staining of pY-STAT3 in 14–3-3ζ knockdown cells. **h** Western blot analysis to qualify pY-STAT3 levels in 14–3-3ζ-overexpressing SK-hep1 cells after being transfected with siHO-1 (or siNC as control) for 48 h

Signal transduction cascades involve multiple enzymes and are orchestrated by selective protein-protein interactions that are essential for the successful progression of intracellular signals [32]. Previous studies have shown that the signaling functions of HO-1 are involved in HO-1 mediated tumor progression [6]. Transcription factor signal arrays analysis and electrophoretic mobility shift assays have demonstrated the increased activation of transcription factors, including STAT3, after transfection with HO-1 [33]. Here, we evaluated whether factors of the STAT3 pathway could be regulated by HO-1 in HCC cell lines. Western blot assay and immunofluorescence staining results showed that the protein level of p-STAT3 (Tyr-705) was significantly reduced upon HO-1 knockdown (Fig. 5a and b), whereas overexpression of HO-1 up-regulated pY-STAT3 levels (Fig. 5c). Moreover, the activity of the luciferase reporter with the STAT3 response element was diminished after HO-1 knockdown in HCC cells under normal culture conditions (Additional file 7: Figure S6a). Consistently, knockdown of HO-1 inhibits expression levels of STAT3 downstream genes, such as survivin, cyclinD1, and MCL1 (Additional file 7: Figure S6b). Interleukin-6 (IL-6) is the most important STAT3 activator and promotes tumor cell proliferation, survival, invasion, angiogenesis [34]. In most HCC cell lines, IL-6 enhances STAT3-Y705 phosphorylation. To further explore the role of HO-1 in modulating STAT3 activation, we next determined whether HO-1 is involved in the IL-6-mediated STAT3 signaling pathway in HCC cells. As shown in Additional file 7: Figure S6c and d, knockdown of HO-1 markedly inhibited IL-6-induced phosphorylation of STAT3 at Y705 and STAT3 activation. These results suggest that HO-1 also mediates IL-6-induced STAT3 activation. We next investigated the molecular mechanisms by which HO-1 regulates STAT3 activity. Overexpression of dominant-negative mutants of STAT3 (STAT3-Y705F) or its upstream components JAK1 (JAK1-K908A) and JAK2 (JAK2-K882A), effectively inhibited IL-6-induced STAT3 activation in reporter assays (Additional file 7: Figure S6e). Interestingly, we found that HO-1-mediated activation of STAT3 could be effectively abolished by mutant of JAK2 (K882A) and STAT3 (Y705F) (Additional file 7: Figure S6f). Since JAK2/STAT3 is required for full phosphorylation of STAT3 after IL-6 stimulation, we investigated whether HO-1 could affect complex formation of JAK2 and STAT3. Coimmunoprecipitation indicated that overexpression of HO-1 promoted the association between JAK2 and STAT3 (Additional file 7: Figure S6g), while its knockdown markedly impaired JAK2–STAT3 association (Additional file 7: Figure S6h). These results suggest that HO-1 mediates the association between JAK2 and STAT3. Collectively, we demonstrated that HO-1 could regulate STAT3 signaling.

The findings that 14–3-3ζ and HO-1 interact in human cancer cells, and that 14–3-3ζ mediates HO-1 stabilization led us to investigate the relative role of each component in regulating STAT3 Tyr705 phosphorylation. Importantly, the increased phosphorylation of STAT3 conferred by HO-1 overexpression could be abolished by 14–3-3ζ knockdown (Fig. 5d). Interestingly, 14–3-3ζ has been reported to regulate STAT3 constitutive activation in multiple myeloma cells [35]. In non-small cell lung cancer (NSCLC), 14–3-3ζ can enhance the phosphorylation of STAT3, which promotes growth and angiogenesis in NSCLC [36]. Thus, we next analyzed whether STAT3 activation was also affected by 14–3-3ζ in HCC cell lines. As expected, the levels of STAT3 Tyr705 phosphorylation were significantly decreased when 14–3-3ζ was knocked-down (Fig. 5e and f), whereas pY-STAT3 levels were increased when 14–3-3ζ was overexpressed (Fig. 5g). Furthermore, we confirmed that HO-1 depletion decreased the phosphorylation of STAT3 in overexpressing 14–3-3ζ HCC cells, compared to cells overexpressing 14–3-3ζ alone (Fig. 5h), demonstrating a significant role of HO-1 in 14–3-3ζ-mediated STAT3 signaling activation. Together, these data indicate the 14–3-3ζ/HO-1 regulation of STAT3 signaling.

### STAT3 pathway is involved in 14–3-3ζ/HO-1 regulation of HCC cell proliferation

**Fig. 6:**
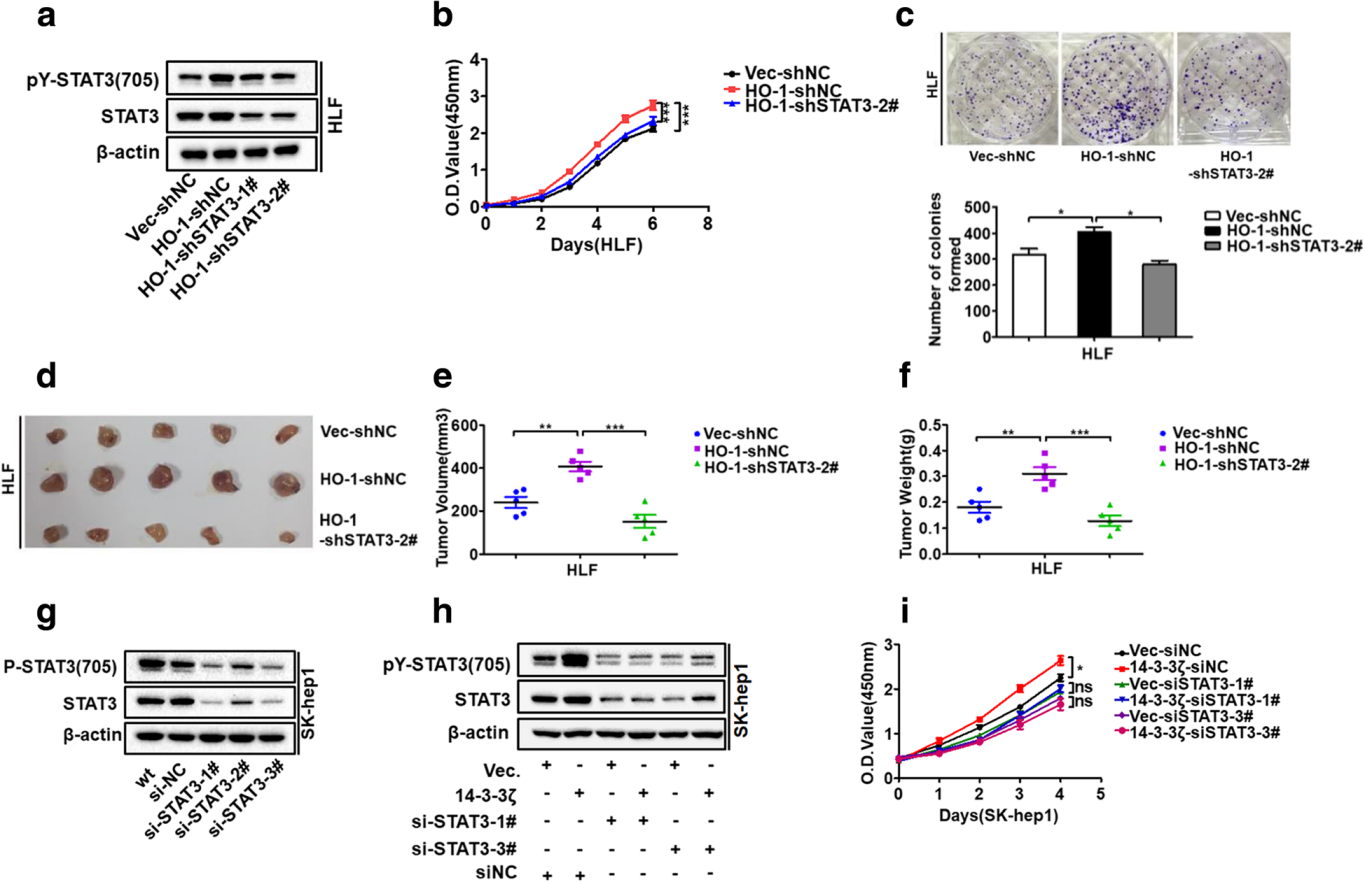
STAT3 pathway is involved in 14–3-3ζ/HO-1 regulation of HCC cell proliferation (**a**) HO-1-overexpressing cells were stably transfected with shNC or shSTAT3 plasmids to generate control (Vec-shNC), HO-1 overexpressing (HO-1-shNC), and HO-1 overexpressing but STAT3 knockdown (HO-1-shSTAT3) stable cells. The pY-STAT3 and total STAT3 levels were examined by western blot analysis. **b** and **c** CCK8 and colony formation assays were performed for indicated cells. **d**-**f** The indicated cells were assessed using a xenograft assay, and tumor growth was measured. **g** si-STAT3 was transfected into SK-hep1 cells to achieve STAT3 knockdown and block phosphorylation of STAT3, as verified using western blot assays. **h** Western blot analysis to quantify pY-STAT3 level in 14–3-3ζ-overexpressing cells after transfection with siSTAT3 (or siNC as control) for 48 h. **i** After transfection with siSTAT3 (or siNC as control) for 48 h, the cells were collected to performed CCK8 assay

Given the essential roles of the STAT3 pathway in the regulation of cell proliferation [37], we evaluated the involvement of the STAT3 pathway in 14–3-3ζ/HO-1 regulation of HCC proliferation. To test this, the effects of HO-1 on proliferation were analyzed in HLF cells with or without STAT3 signaling blocking. Using loss-of-function strategies, we demonstrated that specific shRNA-mediated knock down of STAT3 gene expression could block STAT3 signaling and reverse the HO-1 overexpression-induced cell proliferation (Fig. 6a, b and c) and in vivo tumorigenicity (Fig. 6d-f). The results indicated the effect of HO-1 in promoting HCC proliferation through the STAT3 signaling pathway. We next explored whether 14–3-3ζ-regulated proliferation was also correlated with STAT3 signaling modulation. To test this hypothesis, we utilized loss-of-function strategies with specific siRNAs to block STAT3 signaling in SK-hep1 cells stably overexpressing 14–3-3ζ (Fig. 6g and h). CCK-8 assays revealed that the 14–3-3ζ overexpression-induced increases in cell proliferation capability were delayed by transfection with a STAT3-specific siRNA (Fig. 6i). Taken together, these data further confirmed that 14–3-3ζ/HO-1 axis acts on HCC cell proliferation through the regulation of the STAT3 pathway.

## Discussion

HO-1 is highly induced in various types of cancers [38]. A growing body of evidence supports an emerging model in which HO-1 plays a central role in carcinogenesis and can potentially influence tumor growth in multiple tumor systems, thereby making it an attractive target to prevent both primary and metastatic tumor growth [5]. Substantial work has been carried out to understand how HO-1 expression is regulated, particularly at the transcriptional level [39]. In the promoter region of HO-1, several binding sites are present for different transcription factors that can be activated in oxidative stress conditions like activator protein-1, hypoxia-inducible factor-1, nuclear factor-kappa B, and nuclear factor erythroid 2-related factor 2 (Nrf2) [40]. However, little is known about the factors that regulate HO-1 stability. The present study reports the identification of a physical complex that forms between 14 and 3-3ζ and HO-1. This interaction increases the stability of HO-1. Increased concentration of the partner protein correlated with the elevated levels of HO-1 protein. 14–3-3ζ knockdown-induced reduction in HO-1 protein expression could be rescued by addition of the proteasome inhibitor MG132. Furthermore, HO-1 was stabilized through the 14–3-3ζ-mediated prevention of ubiquitination and degradation.

Increasing evidence in support of the functional importance of HO-1 has stimulated considerable interest in the mechanism underlying the degradation of HO-1. The HO-1 protein is anchored in the ER through its C-terminal transmembrane segment (TMS) that comprises amino acids 266–285, with the remainder of the protein being cytoplasmic [41]. HO-1 undergoes ubiquitination and proteasome-mediated degradation through an ER-associated degradation pathway [7]. Recently, several E3 ligases were shown to be responsible for HO-1 ubiquitination and degradation [7, 8]. Lin et al. identified HO-1 as a physiological substrate of TRC8, an E3 ligase. Moreover, HO-1 lacking its single C-terminal TMS was not co-immunoprecipitated and ubiquitinated by TRC8 [8]. The TMS of HO-1 functions as a membrane anchor and is important in the stabilization and function of HO-1 in the ER [41].

We presently demonstrate that the C-terminal TMS region of HO-1 is essential for the interaction with 14–3-3ζ. With respect to 14–3-3ζ, by interacting with its partners, 14–3-3 proteins could affect various physical and functional aspects of these proteins, such as the binding ability to other proteins, the subcellular distribution, the stability, the catalytic activity, or it may also serve as an adaptor/scaffold protein. More recent studies have indicated that members of the 14–3-3 protein family, such as 14–3-3ζ, and 14–3-3β, are involved in the regulation of ubiquitin-mediated proteasomal degradation of several tumor suppressors or oncogenic proteins [42, 43]. Similarly, 14–3-3 binding can stabilize a number of interacting partners, such as Cdt2 or β-catenin, by impeding its binding to the proteasome system [12, 44]. Moreover, 14–3-3 can regulate the activity of the E3 ubiquitin ligase [23, 45]. 14–3-3 was identified as an interacting partner with some E3 ubiquitin ligases using MS-based quantification and the binding of 14–3-3 could prevent E3 ubiquitin ligase activities, such as TRIM32 autoubiquitylation and transubiquitylation [23]. We cannot rule out the possibility that 14–3-3ζ may enhance HO-1 expression by regulating the degradation of other proteins that affect HO-1 protein stability. Nevertheless, using confocal immunofluorescent analysis, we observed the subcellular localization of HO-1 and 14–3-3ζ and found HO-1 and 14–3-3ζ were localized to ER in tumor cells. HO-1 ubiquitination occurs on ER. We assume that 14–3-3ζ may interact with HO-1 through multiple contacts and act as a cofactor for some specific deubiquitinase or it affects the action of E3 ligases. It is possible that 14–3-3ζ binding regulates HO-1 ubiquitination by inhibiting the E3 ubiquitin ligase activity or altering the conformation of HO-1 and/or obstructs the contact with its E3 ligases for polyubiquitination. These aspects of the mechanism remain to be explored.

Both 14–3-3ζ and HO-1 are associated with cell proliferation [10, 38]. As tumorigenesis is a complex process, it will be of great interest to learn whether or not 14–3-3ζ may also function as a tumor promoter in certain cell contexts. Here, the oncogenic roles of 14–3-3ζ in HCC were investigated. The results indicate that 14–3-3ζ can promote HCC cell proliferation in vitro and accelerate HCC tumor growth in vivo. The question arises as to whether HO-1 stabilization plays a role in these reported functions of the 14–3-3ζ protein. Next, our study found that suppressing HO-1 expression significantly decreased 14–3-3ζ overexpression-enhanced cell proliferation, and growth of xenograft HCC tumors. Therefore, we have revealed a novel mechanism by which 14–3-3ζ promotes HCC tumor growth through HO-1.

STAT3 is a well-known transcriptional activator that regulates the expression of a variety of genes that are critical for cell differentiation, proliferation, apoptosis, angiogenesis, and metastasis and dysregulation of STAT3 activity results in tumorigenesis [37]. Notably, the HO-1 component of the 14–3-3ζ/HO-1 complex has an important role in the STAT3 pathways regulating processes that include inflammation, cell survival, and cell proliferation [33, 46, 47]. In this study, we identified HO-1 as an important mediator of STAT3 activation. Overexpression of HO-1 markedly potentiated IL-6- induced STAT3 activation, whereas HO-1 deficiency had opposite effects. Moreover, knockdown of HO-1 impaired the associations of JAK2 with STAT3, and inhibited transcription levels of STAT3 downstream genes. These results suggest that HO-1 is involved in assembly of JAK2–STAT3 complexes and subsequent phosphorylation of STAT3 following IL-6 stimulation. Then, the present and previous findings lead us to propose a novel role of the 14–3-3ζ/HO-1 axis in the STAT3 signaling pathway. We found that STAT3 phosphorylation was suppressed by knockdown of 14–3-3ζ gene expression. In addition, we demonstrated that 14–3-3ζ knockdown can reverse the upregulation of pY-STAT3 induced by the overexpression of HO-1, whereas HO-1 inhibition can reverse the upregulation of pY-STAT3 induced by the overexpression of 14–3-3ζ. These data indicate the 14–3-3ζ/HO-1 regulation of STAT3 signaling. Our data further suggest that the STAT3 signaling pathway is involved in HO-1-regulated tumor growth of HCC. Moreover, specific siRNA targeting of STAT3 could effectively decrease the levels of pY-STAT3 and abolish 14–3-3ζ-mediated proliferation, implying that the oncogenic function of 14–3-3ζ in HCC is dependent on the STAT3 pathway. Thus, the current findings provide novel mechanistic evidence that the 14–3-3ζ/HO-1 axis acts on HCC cell proliferation through the regulation of the STAT3 pathway.

## Conclusions

Using a combined IP/MS approach, we report for the first time the physical association between 14 and 3-3ζ and HO-1. 14–3-3ζ interacts with HO-1 and stabilizes HO-1 by inhibiting its ubiquitination. Importantly, HO-1 down-regulation significantly suppresses 14–3-3ζ-mediated enhancement of cell proliferation. The STAT3 pathway is involved in 14–3-3ζ/HO-1 regulation of HCC cell proliferation. The findings established the 14–3-3ζ-HO-1-STAT3 axis as an important regulatory mechanism of cancer cell growth and may provide potential targets for HCC prevention and treatment.

## Additional files

Additional file 1: **Table S1.** Primers for shRNA lentiviral constructs; **Table S2.** siRNA target sequences; **Table S3.** The sequences of primers used to generate each mutant; **Table S4.** Sequence of primers for qRT-PCR. (DOC 73 kb)

Additional file 2: **Figure S1.** related to Fig. 1. 14–3-3ζ was identified as HO-1 interactor. (**a**) 14–3-3 protein groups were identified by LC-MS/MS. (**b**) Collision-induced dissociation spectra of 14–3-3ζ peptides identified using gradient elution LC-MS/MS analysis of FLAG-HO-1 immunoprecipitates. (TIF 16429 kb)

Additional file 3: **Figure S2.** (**a**) Immunofluorescence staining for HO-1 localization. HO-1 localization was analysed by immunofluorescence with anti-hmox1 antibody (red). ER compartments and nuclei were stained with ERp72 protein (green) and DAPI (blue), respectively (scale bars, 10 μm). (**b**) Immunofluorescence staining for 14–3-3ζ localization. 14–3-3ζ localization was analysed by immunofluorescence with anti-14-3ζ antibody (red). ER compartments and nuclei were stained with ERp72 protein (green) and DAPI (blue), respectively (scale bars, 10 μm). (**c**) Schematic diagram of point mutants of FLAG-tagged HO-1 used in this study. (**d**) Effect of the S8A, T124A, S247A, and S651A HO-1 mutations on 14–3-3 binding. HEK293 cells were co-transfected with plasmids encoding the indicated Flag-tagged full-length HO-1, or its mutants, as well as HA-14-3-3ζ. The lysates were then immunoprecipitated with an anti-FLAG antibody followed by immunoblotting with indicated antibodies. (TIF 12495 kb)

Additional file 4: **Figure S3.** (**a**, **b**, **c**, **d**) Western blotting (left panel; **a**, **c**) and qRT-PCR (right panel; **b**, **d**) were used to analyze HO-1 knock-down cells, or HO-1 overexpressing cells for protein and mRNA levels of HO-1 and 14–3-3ζ. (**e**) HO-1 knockdown or sh-NC control cells were treated with cycloheximide (CHX) for the indicated times and the expression of endogenous 14–3-3ζ protein was analyzed by western blotting. (**f**) A quantification of 14–3-3ζ protein levels normalized to β-actin and 0 h CHX is shown. Experiments were repeated for three times, and a representative experiment is presented. (**g**) 293 T cells co-transfected with the indicated plasmids were immunoblotted with Flag, HA, and β-actin antibodies. (**h**) Relative mRNA level of Flag-HO-1. 293 T cells co-transfected with the indicated plasmids were used to perform qRT-PCR experiments. (TIF 16080 kb)

Additional file 5: **Figure S4.** (**a**, **b**) qRT-PCR was used to analyze in HCC HLF(a) and Bel7402(b) cells for mRNA levels of 14–3-3 isoforms: 14–3-3ζ, 14–3-3β, 14–3-3ε, 14–3-3γ,14–3-3θ, and 14–3-3η. (**c**-**f**) Real-time PCR(top panel) and Western blot analysis(bottom panel) to respectively quantify mRNA and protein expression of HO-1 after transfection with si14–3-3β, si14–3-3ε, si14–3-3γ,si14–3-3θ, and si14–3-3η (or siNC as control) for 48 h. (TIF 16355 kb)

Additional file 6: **Figure S5.** (**a**, **c**) HCC Bel7402 and SK-hep1 cells with silenced or enhanced 14–3-3ζ expression were grown in normal culture conditions. 48 h later, cell viability was analyzed by Trypan blue exclusion assay and is represented as the mean percentage cell survival of 3 independent experiments (*n* = 3, mean ± SD). (**b**, **d**) HCC Bel7402 and SK-hep1 cells with silenced or enhanced 14–3-3ζ expression were stained with a combination of annexin V and PI and analyzed by FACS. The quantitative of Annexin V-positive cells are shown in right panel. The mean value (mean ± s.d.) of three independent experiments is shown. (**e**) TUNEL staining was performed to detect apoptosis of HCC xenograft tumors derived from shNC and sh14–3-3ζ cells. Scale bars 200 μm. (**f**) The average apoptotic cell counts were calculated on the basis of TUNEL staining. (**g, h**) HO-1-knockdown HLF cells were grown in normal conditions. 48 h later, Cell viability was assessed by Trypan blue exclusion assay (g); Cell apoptosis was assessed with flow cytometric analysis using Annexin V kit (h). Data are presented as mean ± SD from three independent experiments. (TIF 17140 kb)

Additional file 7: **Figure S6.** (a) Luciferase assays for HCC HLF cells transfected with HO-1 siRNAs. (b) Expression of STAT3-targeted genes was examined in small interfering RNA (siHO-1)-transfected-HLF cells by real-time PCR. Glyceraldehyde 3-phosphate dehydrogenase (GAPDH) was used as an endogenous control. (c) HLF shNC and shHO-1 cells were serum starved overnight and treated with 20 ng/ml IL-6 for the indicated time period. Whole-cell lysates were prepared and subject to western blot analysis using the indicated antibodies. (d) Effects of HO-1 knockdown on IL-6-induced activation of STAT3 reporter. HCC HLF shNC and shHO-1 cells were transfected with indicated reporter plasmids. Twenty hours after transfection, cells were treated with IL-6 (20 ng/mL), or left untreated for 12 h in serum-free DMEM before luciferase assays were performed. (e) Effects of dominant-negative mutants of STAT3 (STAT3-Y705F) and its upstream component JAK1 (JAK1-K908A) and JAK2 (JAK2-K882A) on IL-6-induced STAT3 activation. HCC cells were transfected with STAT3 reporter, and the indicated mutant plasmids. Twenty hours after transfection, cells were treated with IL-6 (20 ng/mL), or left untreated for 12 h in serum-free DMEM before luciferase assays were performed. (f) Effects of various dominant-negative mutants on HO-1-mediated STAT3 activation. HCC cells were transfected with STAT3 reporter, HO-1 and the indicated mutant plasmids for 24 h before luciferase assays. (g) Overexpression of HO-1 promotes JAK2–STAT3 interaction. HLF HO-1 overexpressing cells were starved overnight followed by stimulation with IL-6 (20 ng/mL) for 30 min. Coimmunoprecipitation and immunoblot analysis were performed with the indicated antibodies. (h) Knockdown of HO-1 impairs JAK2–STAT3 interaction. The control and HO-1-knockdown Bel7402 cells were starved overnight followed by stimulation with IL-6 (20 ng/mL) for 30 min. Coimmunoprecipitation and immunoblot analysis were performed with the indicated antibodies. (TIF 13369 kb)

## Abbreviations

AKT: Protein Kinase B
CCK-8: Cell counting kit − 8
CHX: Cycloheximide
DMEM: Dulbecco’s Modified Eagle’s Medium
ER: Endoplasmic reticulum
FACS: Fluorescence activated Cell Sorting
FITC: Fluorescein isothiocyanate
GAPDH: glyceraldehyde-3-phosphate dehydrogenase
HCC: Hepatocellular carcinoma
HO-1: Heme oxygenase-1
IL-6: Interleukin-6
IP: Immunoprecipitation
IP/MS: Immunoprecipitation/mass spectrometry
JAK: Janus kinase
mRNA: Messenger ribonucleic acid
Nrf2: Nuclear factor erythroid 2-related factor 2
NSCLC: Non-small cell lung cancer
PBS: Phosphate buffered saline
PI: Propidiumiodide
RT-PCR: Real-time polymerase chain reaction
SDS-PAGE: Sodium dodecyl sulphate-polyacrylamide gel
shRNA: Short hairpin RNA
siRNA: Small interfering RNA
STAT3: Signal transducers and activators of transcription 3
TMS: Transmembrane segment
TRC8: Translocation in renal carcinoma, chromosome 8
TRIM32: Tripartite motif containing 32
