# Supplementary material for: 14–3-3ζ inhibits heme oxygenase-1 (HO-1) degradation and promotes hepatocellular carcinoma proliferation: involvement of STAT3 signaling
Source: J Exp Clin Cancer Res. 2019 Jan 3;38:3. doi: 10.1186/s13046-018-1007-9 (PMC6319010; doi:10.1186/s13046-018-1007-9)
Supplement: Supplementary file 1 — Table S1. Primers for shRNA lentiviral constructs; Table S2. siRNA target sequences; Table S3. The sequences of primers used to generate each mutant; Table S4. Sequence of primers for qRT-PCR. (DOC 73 kb) [file 13046_2018_1007_MOESM1_ESM.doc]

**Table S1.Primers for shRNA lentiviral constructs**

| **shRNA target sequences** | |  |  |
| --- | --- | --- | --- |
| **Gene** | **Symbol** | **Forward(5’ to 3’)** | **Reverse (5’ to 3’)** |
| **HO-1** | **ShHO-1-1#** | CCGGACAGTTGCTGTAGGGCTTTATCTCGAGATAAAGCCCTACAGCAACTGTTTTTTG | AATTCAAAAAACAGTTGCTGTAGGGCTTTATCTCGAGATAAAGCCCTACAGCAACTGT |
|  | **ShHO-1-2#** | CCGGGCTGAGTTCATGAGGAACTTTCTCGAGAAAGTTCCTCATGAACTCAGCTTTTTG | AATTCAAAAAGCTGAGTTCATGAGGAACTTTCTCGAGAAAGTTCCTCATGAACTCAGC |
| **14-3-3ζ** | **sh14-3-3ζ-1#** | CCGGGCAGAGAGCAAAGTCTTCTATCTCGAGATAGAAGACTTTGCTCTCTGCTTTTT | AATTAAAAAGCAGAGAGCAAAGTCTTCTATCTCGAGATAGAAGACTTTGCTCTCTGC |
|  | **sh14-3-3ζ-2#** | CCGGCTCTGTGTTCTATTATGAGATCTCGAGATCTCATAATAGAACACAGAGTTTTT | AATTAAAAACTCTGTGTTCTATTATGAGATCTCGAGATCTCATAATAGAACACAGAG |
| **STAT3** | **shSTAT3-1#** | CCGGGCAAAGAATCACATGCCACTTCTCGAGAAGTGGCATGTGATTCTTTGCTTTTTG | AATTCAAAAAGCAAAGAATCACATGCCACTTCTCGAGAAGTGGCATGTGATTCTTTGC |
|  | **shSTAT3-2#** | CCGGGCACAATCTACGAAGAATCAACTCGAGTTGATTCTTCGTAGATTGTGCTTTTTG | AATTCAAAAAGCACAATCTACGAAGAATCAACTCGAGTTGATTCTTCGTAGATTGTGC |

|  |  |  |
| --- | --- | --- |

**Table S2. The sequences of primers used to generate each mutant**

| **HO-1 mutants** | **Forward(5’ to 3’)** | **Reverse(5’ to 3’)** |
| --- | --- | --- |
| S8A | CGTCCGCAACCCGACGCCATGCCCCAGGATTTG | CAAATCCTGGGGCATGGCGTCGGGTTGCGGACG |
| T124A | CACGAGGTGGGGCGCGCCGAGCCCGAGCTGCTG | CAGCAGCTCGGGCTCGGCGCGCCCCACCTCGTG |
| S188A | CGCTCCCGCATGAACGCCCTGGAGATGACTCCC | GGGAGTCATCTCCAGGGCGTTCATGCGGGAGCG |
| S247A | AACAAAGTGCAAGATGCCGCCCCCGTGGAGACT | AGTCTCCACGGGGGCGGCATCTTGCACTTTGTT |
| 1-264aa | ATGGAGCGTCCGCAACCCGACAG | CTGGGAGCGGGTGTTGAGTGGG |
| 1-221aa | ATGGAGCGTCCGCAACCCGACAG | CAGCAGCTCCTGCAACTCCTCA |
| 1-119aa | ATGGAGCGTCCGCAACCCGACAG | GTGGAGCCGCTTCACATAGCGC |
| 119-288aa | GAGGTGGGGCGCACAGAGCCCGA | CATGGCATAAAGCCCTACAGCA |

**Table S3. siRNA target sequences**

| **RNAi target sequences** |  |  |
| --- | --- | --- |
| **Gene** | **Symbol** | **Sequence** |
|  |  |  |
| **HO-1** | siHO-1-1 | CGTTCCTGCTCAACATCCA |
|  | siHO-1-2 | CGATGGGTCCTTACACTCA |
|  | siHO-1-3 | CAGTTGCTGTAGGGCTTTA |
| **STAT3** | siSTAT3-1 | GCCTCTCTGCAGAATTCAA |
|  | siSTAT3-2 | AGTCAGGTTGCTGGTCAAA |
|  | siSTAT3-3 | CCGTGGAACCATACACAAA |
| **14-3-3β** | si14-3-3β-1 | CGCTGAATGAAGAGTCTTATA |
|  | si14-3-3β-2 | CCTCCTTTCTAGCTGAACAAA |
| **14-3-3ε** | si14-3-3ε-1 | GCTGACAGTTGAAGAAAGAAA |
|  | si14-3-3ε-1 | CGACGAAATGGTGGAGTCAAT |
| **14-3-3γ** | si14-3-3γ-1 | CCTGCTGGATAACTACCTGAT |
|  | si14-3-3γ-2 | CCCAAGTAATTGTAGGAAGAT |
| **14-3-3θ** | si14-3-3θ-1 | CAGTTGCTTAGAGACAACCTA |
|  | si14-3-3θ-2 | CAAACGATAGATAATTCCCAA |
| **14-3-3η** | si14-3-3η-1 | AGAAATTGGAGAAAGTTAAAG |
|  | si14-3-3η-2 | TGGACACACTAAACGAGGATT |
|  |  |  |

**Table S4. Sequence of primers for qRT-PCR**

| **Gene** | **Forward(5’ to 3’)** | **Reverse(5’ to 3’)** |
| --- | --- | --- |
| **HO-1** | AAGACTGCGTTCCTGCTCAAC | AAAGCCCTACAGCAACTGTCG |
| **14-3-3ζ** | CCTGCATGAAGTCTGTAACTGAG | GACCTACGGGCTCCTACAACA |
| **14-3-3ε** | GATTCGGGAATATCGGCAAATGG | GCTGGAATGAGGTGTTTGTCC |
| **14-3-3β** | CATGAAGGCAGTCACAGAACA | CTCACGGTACTCTTTGCCCAT |
| **14-3-3γ** | AGCCACTGTCGAATGAGGAAC | CTGCTCAATGCTACTGATGACC |
| **14-3-3θ** | AGGGTCATCTCTAGCATCGAG | CCACTTTCTCCCGATAGTCCTT |
| **14-3-3η** | GACATGGCCTCCGCTATGAAG | ATGCTGCTAATGACCCTCCAG |
| **cyclinD1** | GGCGGATTGGAAATGAACTT | TCCTCTCCAAAATGCCAGAG |
| **survivin** | AGGACCACCGCATCTCTACAT | AAGTCTGGCTCGTTCTCAGTG |
| **MCL1** | CATTCCTGATGCCACCTTCT | TCGTAAGGACAAAACGGGAC |
| **GAPDH** | GGGGCTCTCCAGAACATCATCC | ACGCCTGCTTCACCACCTCTT |
