# Supplementary figures and images for: 14–3-3ζ inhibits heme oxygenase-1 (HO-1) degradation and promotes hepatocellular carcinoma proliferation: involvement of STAT3 signaling
Source: J Exp Clin Cancer Res. 2019 Jan 3;38:3. doi: 10.1186/s13046-018-1007-9 (PMC6319010; doi:10.1186/s13046-018-1007-9)

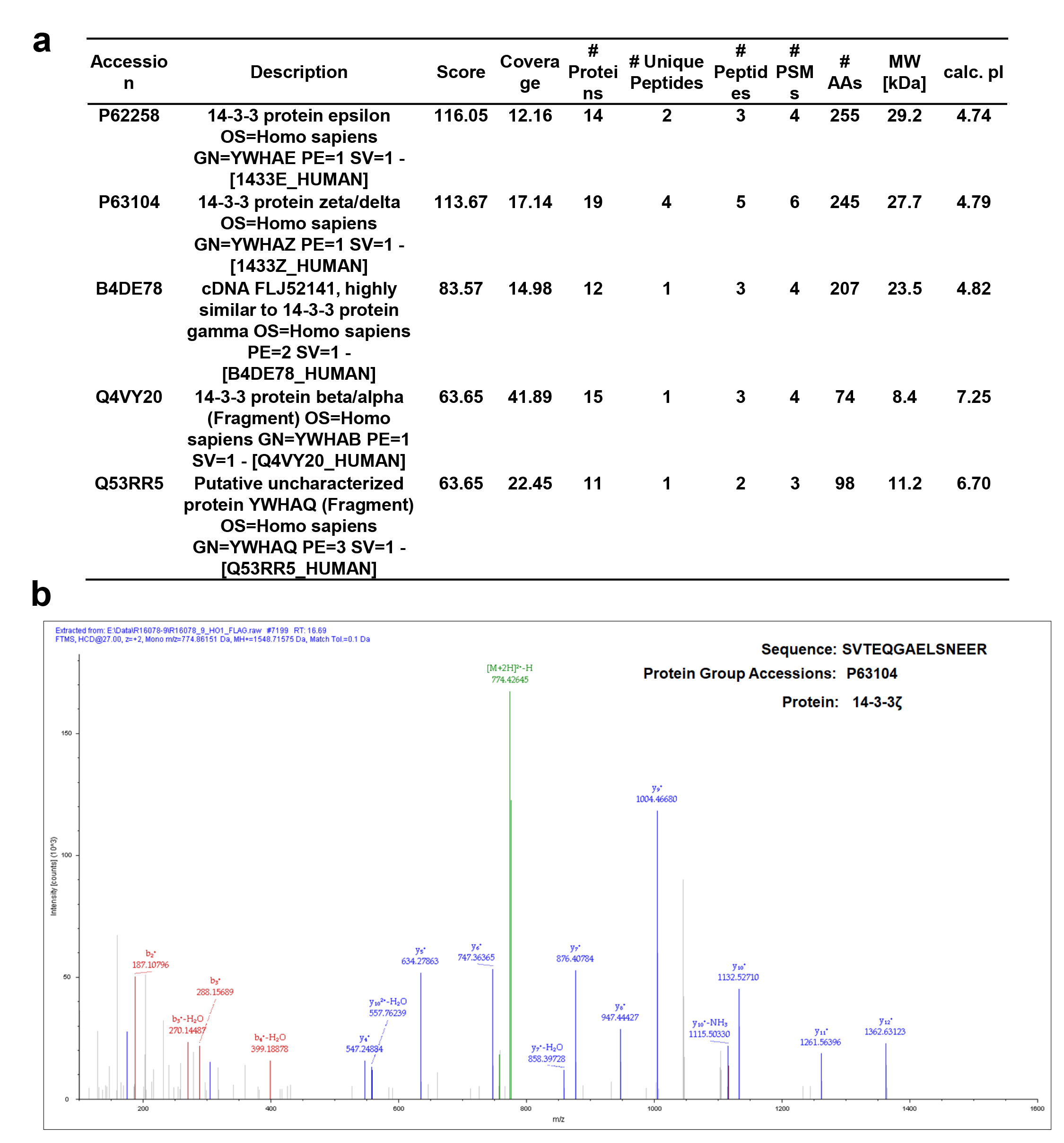

Supplement: Supplementary file 2 — Figure S1. related to Fig. 1. 14–3-3ζ was identified as HO-1 interactor. (a) 14–3-3 protein groups were identified by LC-MS/MS. (b) Collision-induced dissociation spectra of 14–3-3ζ peptides identified using gradient elution LC-MS/MS analysis of FLAG-HO-1 immunoprecipitates. (TIF 16429 kb) [file 13046_2018_1007_MOESM2_ESM.tif]

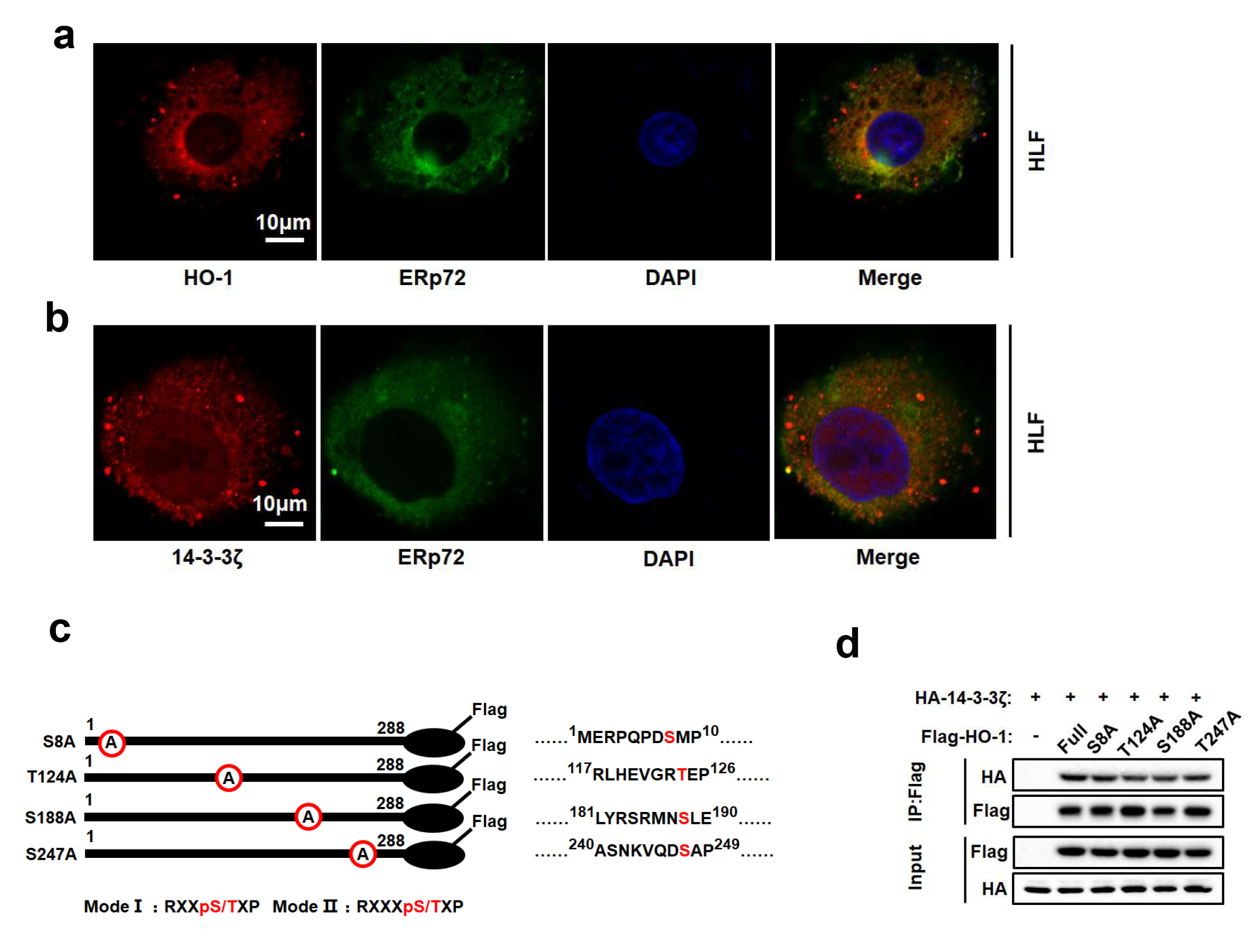

Supplement: Supplementary file 3 — Figure S2. (a) Immunofluorescence staining for HO-1 localization. HO-1 localization was analysed by immunofluorescence with anti-hmox1 antibody (red). ER compartments and nuclei were stained with ERp72 protein (green) and DAPI (blue), respectively (scale bars, 10 μm). (b) Immunofluorescence staining for 14–3-3ζ localization. 14–3-3ζ localization was analysed by immunofluorescence with anti-14-3ζ antibody (red). ER compartments and nuclei were stained with ERp72 protein (green) and DAPI (blue), respectively (scale bars, 10 μm). (c) Schematic diagram of point mutants of FLAG-tagged HO-1 used in this study. (d) Effect of the S8A, T124A, S247A, and S651A HO-1 mutations on 14–3-3 binding. HEK293 cells were co-transfected with plasmids encoding the indicated Flag-tagged full-length HO-1, or its mutants, as well as HA-14-3-3ζ. The lysates were then immunoprecipitated with an anti-FLAG antibody followed by immunoblotting with indicated antibodies. (TIF 12495 kb) [file 13046_2018_1007_MOESM3_ESM.tif]

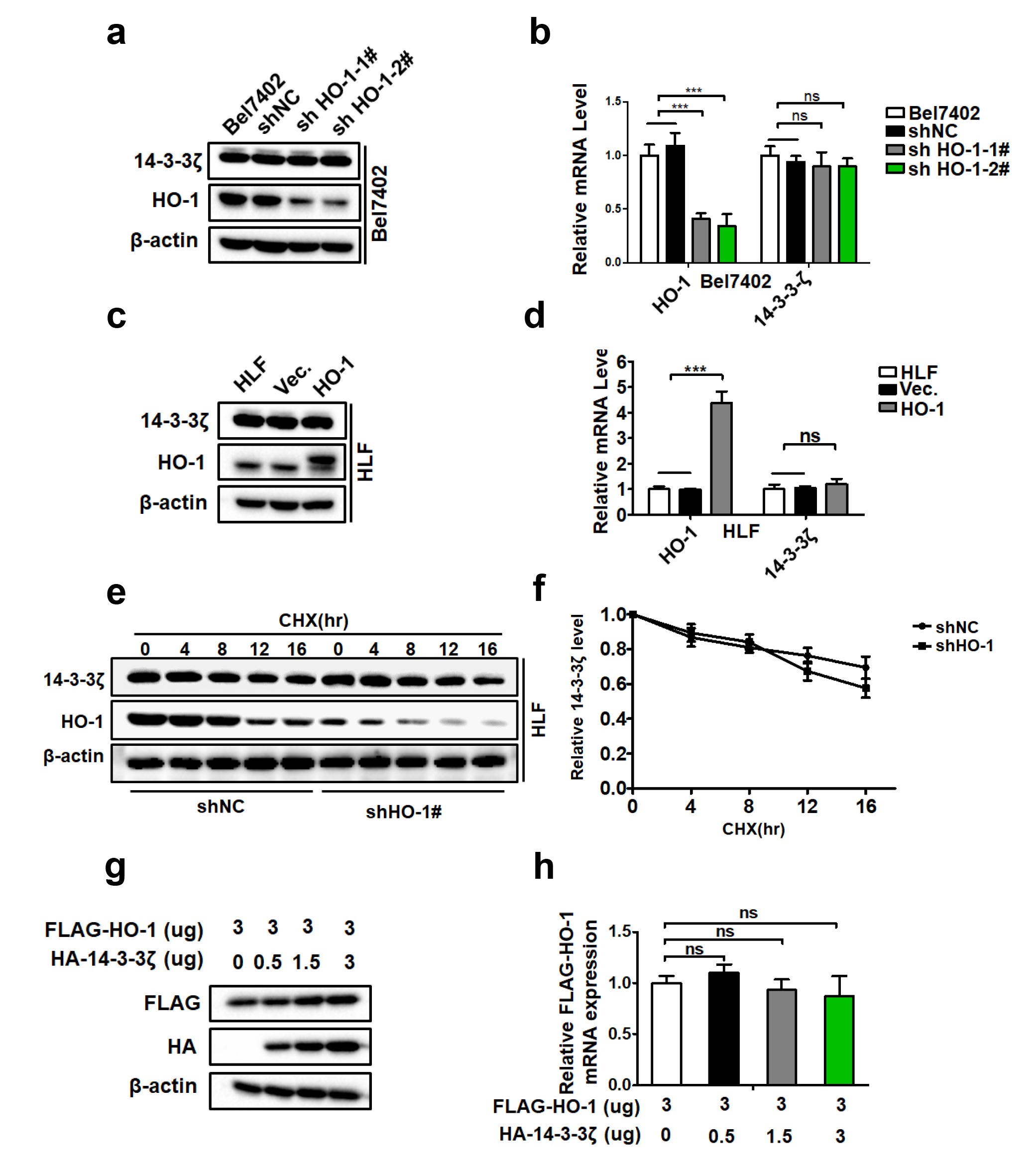

Supplement: Supplementary file 4 — Figure S3. (a, b, c, d) Western blotting (left panel; a, c) and qRT-PCR (right panel; b, d) were used to analyze HO-1 knock-down cells, or HO-1 overexpressing cells for protein and mRNA levels of HO-1 and 14–3-3ζ. (e) HO-1 knockdown or sh-NC control cells were treated with cycloheximide (CHX) for the indicated times and the expression of endogenous 14–3-3ζ protein was analyzed by western blotting. (f) A quantification of 14–3-3ζ protein levels normalized to β-actin and 0 h CHX is shown. Experiments were repeated for three times, and a representative experiment is presented. (g) 293 T cells co-transfected with the indicated plasmids were immunoblotted with Flag, HA, and β-actin antibodies. (h) Relative mRNA level of Flag-HO-1. 293 T cells co-transfected with the indicated plasmids were used to perform qRT-PCR experiments. (TIF 16080 kb) [file 13046_2018_1007_MOESM4_ESM.tif]

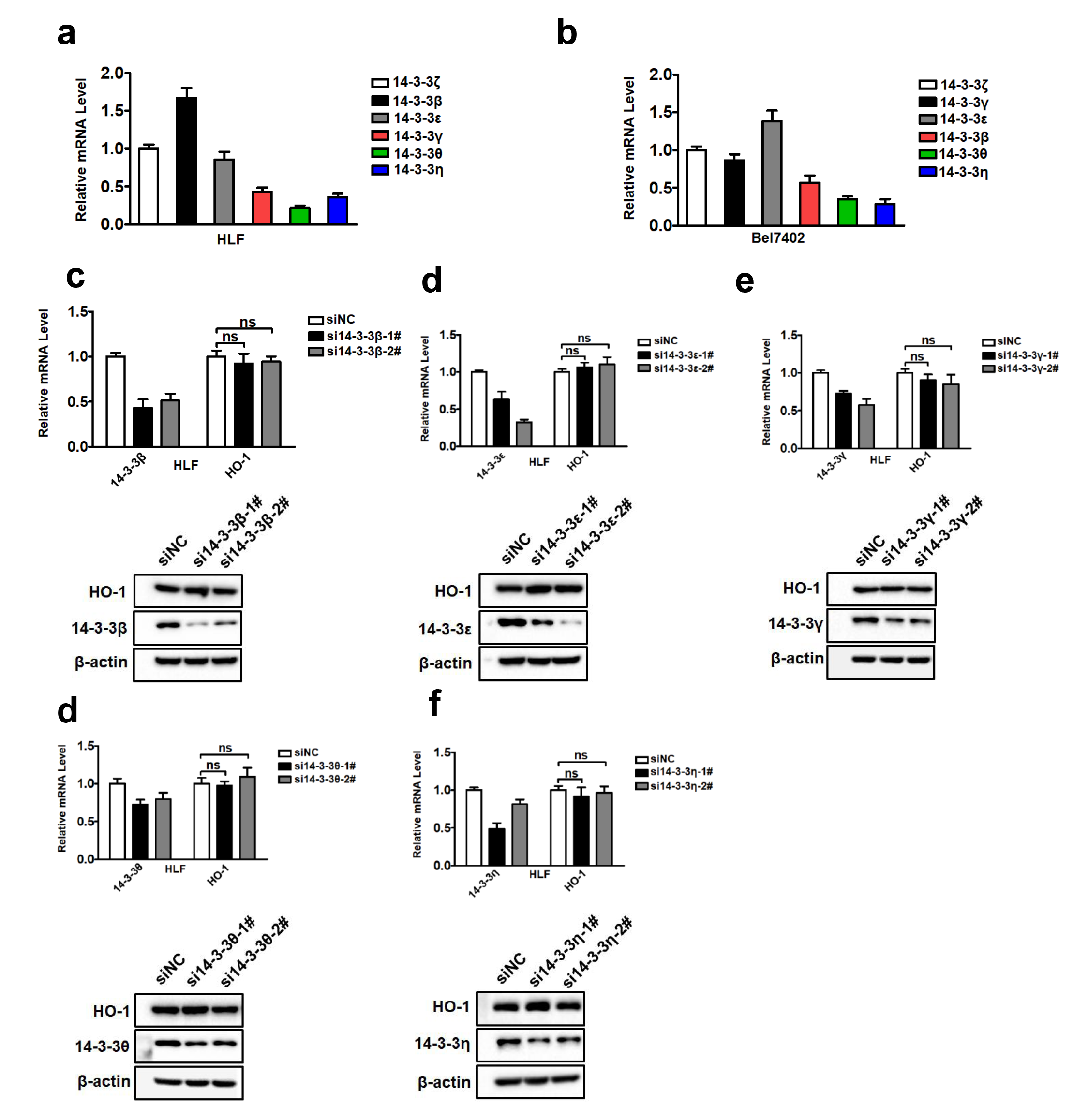

Supplement: Supplementary file 5 — Figure S4. (a, b) qRT-PCR was used to analyze in HCC HLF(a) and Bel7402(b) cells for mRNA levels of 14–3-3 isoforms: 14–3-3ζ, 14–3-3β, 14–3-3ε, 14–3-3γ,14–3-3θ, and 14–3-3η. (c-f) Real-time PCR(top panel) and Western blot analysis(bottom panel) to respectively quantify mRNA and protein expression of HO-1 after transfection with si14–3-3β, si14–3-3ε, si14–3-3γ,si14–3-3θ, and si14–3-3η (or siNC as control) for 48 h. (TIF 16355 kb) [file 13046_2018_1007_MOESM5_ESM.tif]

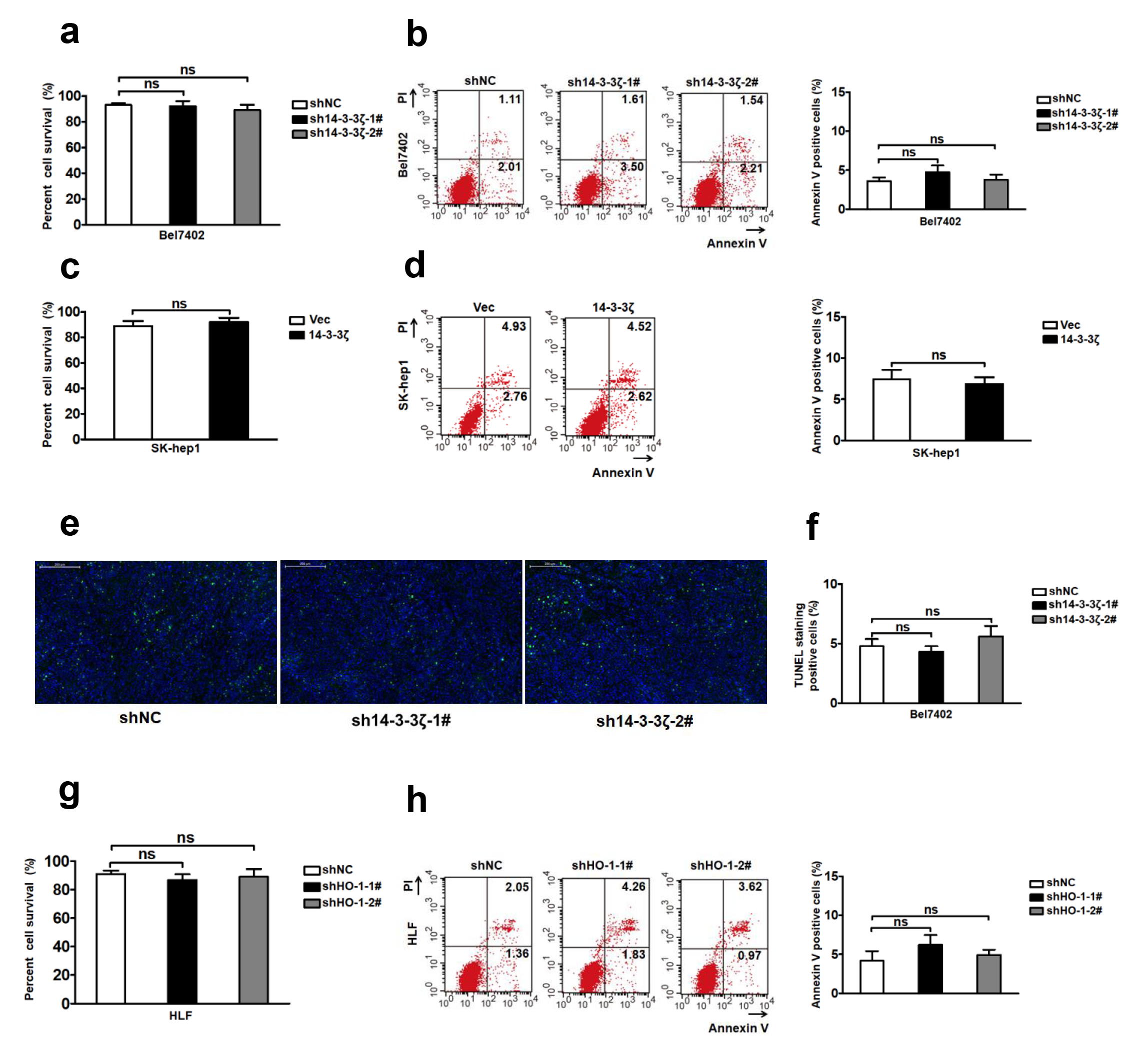

Supplement: Supplementary file 6 — Figure S5. (a, c) HCC Bel7402 and SK-hep1 cells with silenced or enhanced 14–3-3ζ expression were grown in normal culture conditions. 48 h later, cell viability was analyzed by Trypan blue exclusion assay and is represented as the mean percentage cell survival of 3 independent experiments (n = 3, mean ± SD). (b, d) HCC Bel7402 and SK-hep1 cells with silenced or enhanced 14–3-3ζ expression were stained with a combination of annexin V and PI and analyzed by FACS. The quantitative of Annexin V-positive cells are shown in right panel. The mean value (mean ± s.d.) of three independent experiments is shown. (e) TUNEL staining was performed to detect apoptosis of HCC xenograft tumors derived from shNC and sh14–3-3ζ cells. Scale bars 200 μm. (f) The average apoptotic cell counts were calculated on the basis of TUNEL staining. (g, h) HO-1-knockdown HLF cells were grown in normal conditions. 48 h later, Cell viability was assessed by Trypan blue exclusion assay (g); Cell apoptosis was assessed with flow cytometric analysis using Annexin V kit (h). Data are presented as mean ± SD from three independent experiments. (TIF 17140 kb) [file 13046_2018_1007_MOESM6_ESM.tif]

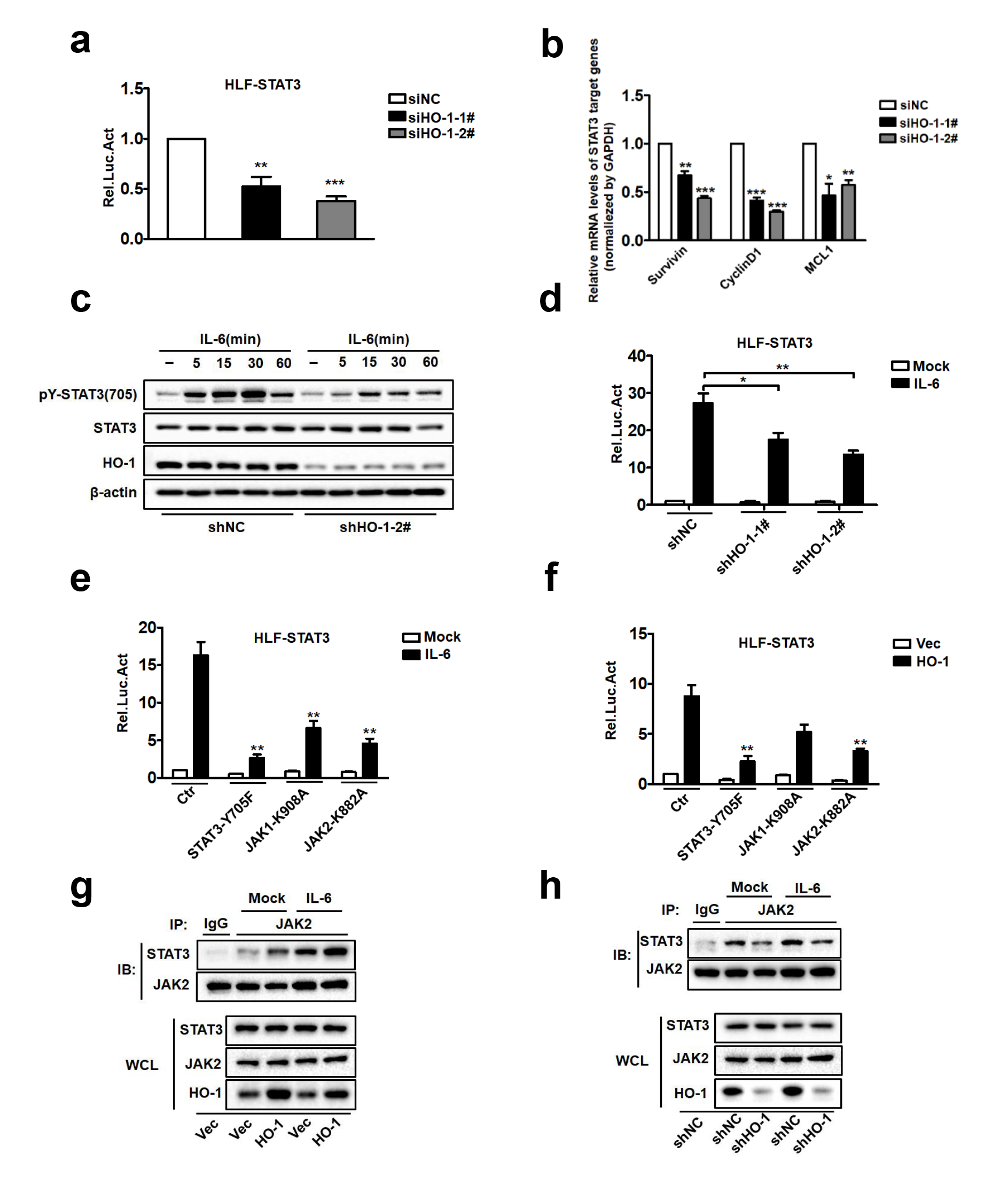

Supplement: Supplementary file 7 — Figure S6. (a) Luciferase assays for HCC HLF cells transfected with HO-1 siRNAs. (b) Expression of STAT3-targeted genes was examined in small interfering RNA (siHO-1)-transfected-HLF cells by real-time PCR. Glyceraldehyde 3-phosphate dehydrogenase (GAPDH) was used as an endogenous control. (c) HLF shNC and shHO-1 cells were serum starved overnight and treated with 20 ng/ml IL-6 for the indicated time period. Whole-cell lysates were prepared and subject to western blot analysis using the indicated antibodies. (d) Effects of HO-1 knockdown on IL-6-induced activation of STAT3 reporter. HCC HLF shNC and shHO-1 cells were transfected with indicated reporter plasmids. Twenty hours after transfection, cells were treated with IL-6 (20 ng/mL), or left untreated for 12 h in serum-free DMEM before luciferase assays were performed. (e) Effects of dominant-negative mutants of STAT3 (STAT3-Y705F) and its upstream component JAK1 (JAK1-K908A) and JAK2 (JAK2-K882A) on IL-6-induced STAT3 activation. HCC cells were transfected with STAT3 reporter, and the indicated mutant plasmids. Twenty hours after transfection, cells were treated with IL-6 (20 ng/mL), or left untreated for 12 h in serum-free DMEM before luciferase assays were performed. (f) Effects of various dominant-negative mutants on HO-1-mediated STAT3 activation. HCC cells were transfected with STAT3 reporter, HO-1 and the indicated mutant plasmids for 24 h before luciferase assays. (g) Overexpression of HO-1 promotes JAK2–STAT3 interaction. HLF HO-1 overexpressing cells were starved overnight followed by stimulation with IL-6 (20 ng/mL) for 30 min. Coimmunoprecipitation and immunoblot analysis were performed with the indicated antibodies. (h) Knockdown of HO-1 impairs JAK2–STAT3 interaction. The control and HO-1-knockdown Bel7402 cells were starved overnight followed by stimulation with IL-6 (20 ng/mL) for 30 min. Coimmunoprecipitation and immunoblot analysis were performed with the indicated antibodies. (TIF 13369 kb) [file 13046_2018_1007_MOESM7_ESM.tif]
